# Meeting Abstracts of the American Society for Exosomes and Microvesicles 2020 Annual Meeting

**DOI:** 10.20517/evcna.2020.10

**Published:** 2020-12-30

**Authors:** Alissa M. Weaver, Ryan P. McNamara, Y Peng Loh, Hannah M. McMillan, Blanca V. Rodriguez, James Erickson, Heather Branscome, Sarah Al Sharif, Ribhav Mishra, Kathleen M Lennon, Amy H. Lee, Jean C. Lee, Bridget Ratitong, Jessica Pullan, Joshua L. Hood, Pooja Khatkar, Janos Zempleni, Yi Zhao, Nicole Comfort, Jordan Pavlic, Nicole Noren Hooten, Sarah F. Andres, Lisa Meyer, Shivani Sharma, Johnny Akers, Laura Perin, Emma Purcell, Dilorom Sass, Tatsuo Kurihara, Shamba Gupta, Ikuhiko Nakase, Maria Cowen, Yuriy Kim, Ryan Reshke, Tsuneya Ikezu, Hameeda Sultana, Killian P. O’Brien, Chukwumaobim D. Nwokwu, Inge S. Zuhorn, Simon Carding, Dennis A. Steindler, Frederik J. Verweij, Pieter Vader, Martin Olivier, Crislyn D’Souza-Schorey, Moran Amit

*The Abstract of the American Society for Exosomes and Microvesicles 2020 Annual Meeting, November 16-19, 2020 [Table 1]

**Table 1:** Table of Content

No.	Abstract Title	Authors	Pages
1	Argonautes in extracellular vesicles: artifact or selected cargo?	Alissa M. Weaver	22
2	Super-resolution microscopy-based resolving of membrane-associated proteins on extracellular vesicles	Ryan P. McNamara, Yijun Zhou Meredith G. Chambers, Anthony. B. Eason, Justin T. Landis, Brian Yang, Blossom A. Damania, Dirk P. Dittmer	23
3	Exosomal carboxypeptidase econfers and CPE-shRNA loaded exosomes inhibit tumorigenesis	Sangeetha Hareendran, Bassam Albraidy, Xuyu Yang, Aiyi Liu, Anne Breggia, Clark C Chen, Y Peng Loh	23
4	Bacterial vesicles: vehicles for Inter-Kingdom communication and modulators of plant immune response	Hannah M. McMillan, Sophia G. Zebell, Jean B. Ristaino, Xinnian Dong, Meta J. Kuehn	24
5	Staphylococcus aureus secretes immunomodulatory RNA via extracellular membrane vesicles	Blanca V. Rodriguez, Meta J. Kuehn	25
6	Separation of EVs from virions in coronavirus infections	James Erickson, Maria Cowen, Yuriy Kim, Anoop Pal, Heather Branscome, Archana Gupta, Fatah Kashanchi	25
7	Use of stem cell extracellular vesicles as a holistic approach towards CNS repair	Heather Branscome, Siddhartha Paul, Dezhong Yin, Weidong Zhou, Lance A. Liotta, Fatah Kashanchi	26
8	Extracellular vesicles from HTLV-1 infected cells regulate viral spread and pathogenesis	Sarah Al Sharif, Daniel O. Pinto, Gifty Mensah, Maria Cowen, Pooja Khatkar, James Erickson, Heather Branscome, Catherine De Marino, Weidong Zhou, Lance A. Liotta, Fatah Kashanchi	27
9	Proteomics of cerebellar exosomal proteins: therapeutic and biomarker implications in spinocerebellar ataxia-1	Ribhav Mishra, Puneet Opal	28
10	Advancing extracellular vesicle characterization with quantitative single molecule localization microscopy	Kathleen M Lennon, Adam L Maddox, Andras Saftics, Matthew S Brehove, Devin L Wakefield, Saumya Das, Kendall Van Keuren-Jensen, Gagandeep Singh, Tijana Jovanovic-Talisman	29
11	Hypocalcemic-induced exosome secretion drives differential metastatic progression of epithelial ovarian cancer	Amy H. Lee, Deepraj Ghosh, Nhat Quach, Michelle R. Dawson	29
12	Orchestration of human macrophage NLRP3 inflammasome activation by Staphylococcus aureus extracellular vesicles	Jean C. Lee, Xiaogang Wang	30
13	Beta-glucan stimulated neutrophils secrete IL-1α through exosomes	Bridget Ratitong, Michaela E. Marshall, Eric Pearlman	31
14	Hypoxia-responsive bovine milk exosomes as targeted carrier for chemotherapeutics	Jessica Pullan, Li Feng, Kaitlin Dailey, Sangeeta Bhallamudi, James Froberg, Lina Alhalhooly, Amanda Brooks, Sathish Venkatachalem, Yongki Choi, Sanku Mallik	31
15	Liver tumor-derived exosomes (small extracellular vesicles) induce macrophage polarity	Mackenzie J. Burroughs, Gina T. Bardi, Joshua L. Hood	32
16	Small RNA-binding molecules inhibit HIV-1 transcription in CNS latent reservoirs	Pooja Khatkar, Maria Cowen, Catherine DeMarino1, Fardokht A Abulwerdi, Lance A Liotta, Stuart F.J Le Grice, Fatah Kashanchi	32
17	Extracellular vesicles and microRNAs in maternal milk are important for growth and gut health during weaning in murine pups	Janos Zempleni. Mahrou Sadri, Fang Zhou	33
18	High-capacity membranes for simple, rapid extracellular vesicle isolation with high yield and purity	Yi Zhao, Brenda Huang, Boris Levitan, Michael Haugwitz, Andrew A. Farmer	34
19	Isolation and characterization of extracellular vesicles in saliva of children with asthma	Nicole Comfort, Tessa Bloomquist, Amparito Cunningham, Marissa Hauptman, Wanda Phipatanakul, Andrea Baccarelli	35
20	Immunoregulatory properties of cancer stem cell derived extracellular vesicles	Jordan Pavlic, Joehleen Archard, Jan Nolta, Johnathon D. Anderson	36
21	Extracellular vesicles in diabetes mellitus carry inflammatory cargo that affects cellular behavior	Nicole Noren Hooten, Sharon F. Wu, David W. Freeman, Nicolle A. Mode, Alan B. Zonderman, Michele K. Evans	36
22	IMP1 modulates extracellular vesicle production in the GI tract through regulation of endosome and autophagy pathways	Sarah F. Andres, Madeline Kuhn, Ranjan Preet, Aurora Blucher, John Favate, Sukanya Das, Shun Liang, Louis Parham, Priya Chatterji, Wei Zhang, Jiegang Yang, Kathryn E. Hamilton, Premal Shah, Gordon Mills, Dan A. Dixon, Wei Guo, Anil K. Rustgi	37
23	Simple workflow for isolation and Western blot detection of MISEV-recommended EV protein-markers	Lisa Meyer, Daniel Enderle, Carsten Lück, Anne Bicke, Yasef Khan, Chris Heger, Martin Schlumpberger, Markus Sprenger-Haussels, Johan Skog, Mikkel Noerholm	38
24	Nanomechanical fingerprinting of single extracellular vesicles	Shivani Sharma	39
25	Development of non-invasive clinically applicable in vivo tracking of extracellular vesicles using magnetic resonance imaging	Johnny Akers, Paola Aguiari, Hasmik Soloyan, Seda Mkhitaryan, Gevorg Karapetyan, Laura Perin, Mya Thu, and Sargis Sedrakyan	39
26	Glomerular heterogeneity and modulation of miR-93-5p: the role of extracellular vesicles	Charmi Dedhia, Paola Aguiari, Hasmik Soloyan, Roger E De Filippo, Sargis Sedrakyan, Laura Perin	40
27	Use of high-capacity membranes for simple, rapid exosome isolations with high yield and purity	Yi Zhao, Boris Levitan, Brenda Huang, Micheal Haugwitz, Andrew Farmer	41
28	Detection of EGFR mutations in extracellular vesicle RNA and protein corresponds to disease status in metastatic lung cancer patients	Emma Purcel, Sarah Owen, Emily Prantzalos, Abigail Radomski, Mina Zeinali, Nayri Carman, Nithya Ramnath, Sunitha Nagrath	41
29	The role of extracellular vesicles in cancer-related fatigue	Dilorom Sass, Wendy Fitzgerald, Leorey Saligan, Leonid Margolis	42
30	Mechanistic analysis of protein transport to extracellular membrane vesicles of a hypervesiculating bacterial strain, Shewanella vesiculosa HM13	Tatsuo Kurihara, Kouhei Kamasaka, Chen Chen, Fumiaki Yokoyama, Tomoya Imai, Takuya Ogawa, Jun Kawamoto	43
31	Mycobacterial dynamin like proteins are necessary for extracellular vesicle release in M. tuberculosis	Shamba Gupta, Ainhoa Palacios, Atul Khataokar, Madhuri Bhagavathula, Matthew B. Neiditch, Padmini Salgame, Rafael Prados-Rosales, G. Marcela Rodríguez	43
32	Intracellular delivery methods using biofunctional peptide-modified extracellular vesicles	Ikuhiko Nakase	44
33	Effects of marijuana on viral transcription in HIV-1 infected cells and resulting extracellular vesicle release	Catherine De Marino, Maria Cowen, Bianca Cotto, Sara Jane Ward, Ronald Tuma, Prasun Datta, Leah H. Rubin, Dianne Langford, Fatah Kashanchi	45
34	Extracellular vesicles release from infected cells prior to virion release	Yuriy Kim, Daniel Pinto, Gifty Mensah, Maria Cowen, James Erickson, Renaud Mahieux, Fatah Kashanchi	45
35	Separation of EVs from virions in coronavirus infections	James Erickson, Maria Cowen, Yuriy Kim, Anoop K. Pal, Heather Branscome, Archana Gupta, Fatah Kashanchi	46
36	Reduction of the therapeutic dose of silencing RNA by packaging it in extracellular vesicles via a pre-microRNA backbone	Ryan Reshke, James A. Taylor, Alexandre Savard, Huishan Guo, Luke H. Rhym, Piotr S. Kowalski, My Tran Trung, Charles Campbell,Daniel G. Anderson, Derrick Gibbings	47
37	Cell type-specific and disease-associated protein networks in extracellular vesicles isolated from human iPSC-derived neural cells and Alzheimer’s disease brain tissues	Yang You, Satoshi Muraoka, Mark P. Jedrychowski, Jianqiao Hu, Amanda K. McQuade, Tracy Young-Pearse, Roshanak Aslebagh, Scott A. Shaffer, Mathew Blurton-Jones, Wayne W. Poon, Steven P. Gygi, Tsuneya Ikezu	48
38	Exosomes mediate Zika virus transmission through SMPD3 neutral Sphingomyelinase in cortical neurons	Hameeda Sultana	49
39	Understanding Intracellular Fate of EV-delivered Content	Killian P. O’Brien, Stefano Ughetto, Xandra O. Breakefield	49
40	Smart Microprobes Imbued with Recognition Element as a Sensitive Bioanalysis Platform for Exosomes	Chukwumaobim D. Nwokwu, Saif Mohammad Ishraq Bari, Gergana G. Nestorova	50
41	Extracellular vesicles for drug delivery to the brain	Bhagyashree S. Joshi, Marit A. De Beer, Ben N.G. Giepmans, Sameh A. Youssef, Alain de Bruin, Harm H. Kampinga, Inge S. Zuhorn	50
42	The uptake, trafficking, and biodistribution of Bacteroides thetaiotaomicron (Bt) generated outer membrane vesicles	Simon Carding	51
43	Infectious Exosomes/Microvesicles in Degenerative and Neoplastic Stem Cell Pathologies	Dennis A. Steindler	52
44	Tracking exosomes in vitro and in vivo	Frederik J. Verweij, Maarten Bebelman, Graça Raposo, D. Michiel Pegtel, Guillaume van Niel	53
45	A CRISPR-Cas9-based reporter system for single-cell detection of extracellular vesicle mediated functional transfer of RNA	Olivier G. de Jong, Daniel E. Murphy, Imre Mäger, Eduard Willms, Antonio GarciaGuerra, Jerney J. Gitz-Francois, Juliet Lefferts, Dhanu Gupta, Sander C. Steenbeek, Jacco van Rheenen, Samir El Andaloussi, Raymond M. Schiffelers, Matthew J. A. Wood, Pieter Vader	54
46	Exploitation of the Leishmania Exosomal Pathway by Leishmania RNA virus 1	Martin Olivier	54
47	Delivery of pre-miRNA Cargo to Tumor Microvesicles	Crislyn D’Souza-Schorey, James Clancy, Ye Zhang	55
48	Head and Neck Cancer Exosomes Drive MicroRNA-mediated Reprogramming of Local Neurons	Patrick J. Hunt, Moran Amit	56
