# Supplementary material for: Meeting Abstracts of the American Society for Exosomes and Microvesicles 2020 Annual Meeting
Source: Extracell Vesicles Circ Nucl Acids. 2020 Dec 30;1(1):20–56. doi: 10.20517/evcna.2020.10 (PMC11648469; doi:10.20517/evcna.2020.10)
Supplement: Related texts

# Other: 1. Argonautes in extracellular vesicles: artifact or selected cargo?

**Authors:** Alissa M. Weaver

**Affiliations:** Department of Cell and Developmental Biology, Vanderbilt University School of Medicine, Nashville, TN, USA.

## Abstract

Argonaute 2 (Ago2) is the essential component of the RNA-Induced Silencing Complex (RISC) that binds miRNAs and promotes mRNA degradation. Extracellular vesicle (EV)-carried miRNAs have been shown to influence gene expression and functional phenotypes in recipient cells. Many investigators have found Ago2 in EVs and it is postulated that Ago2 is a major transporter of miRNAs into small EVs (SEVs), such as exosomes. Others have reported that extracellular Ago2 is non- vesicular. I will discuss issues that may lead to diverging results and show data on the role of culture conditions in the detection of Ago2 in SEVs.

# Other: 2. Super-resolution microscopy-based resolving of membrane-associated proteins on extracellular vesicles

**Authors:** Ryan P. McNamara, Yijun Zhou, Meredith G. Chambers, Anthony. B. Eason, Justin T. Landis, Brian Yang, Blossom A. Damania, Dirk P. Dittmer

**Affiliations:** ^1^Department of Microbiology and Immunology, The University of North Carolina at Chapel Hill, Chapel Hill, NC, USA.; ^2^Lineberger Comprehensive Cancer Center, The University of North Carolina at Chapel Hill, Chapel Hill, NC, USA.; ^*^Presenting Author.

## Abstract

Extracellular vesicles (EV) are secreted from most cell types and are intimately involved in homeostasis. Given that their size can drop well beneath the limit of diffraction of typical light microscopy [< 250 nanometers (nm)], direct visualizations and characterizations of single EV have proven daunting. Conventional approaches to their visualization include light scattering methods such as nanoparticle tracking analysis, transmission and scanning electron microscopy (EM), and highly sensitive nanoscale flow cytometry. Super-resolution microscopy, such as direct stochastic optical reconstruction microscopy (dSTORM) has also been recently employed to view EV. Here, we show that dSTORM can be employed in multiple channels to resolve membrane-associated proteins to nanometer precision. Our dSTORM- based characterizations of EV typically had resolutions of ± 20 nm on the XY axis using excitation lasers of 473 and 640 nm. Size distributions of EV were consistent with other methods of measurement such as light scattering and resistive pulse sensing and were independent of the excitation laser. Moreover, we were able to resolve tetraspanins on the membrane of an individual EV, both by conjugation to- mCherry or through indirect antibody-mediated detection. In conclusion, we have rendered individual EV to nanometer detail through super-resolution microscopy and confirmed the presence of tetraspanin molecules on their surface. We propose that a highly sensitive analysis like this could be adapted to rapidly scan for rare- event biomarkers of disease through fluorescence-based machine learning.

# Other: 3. Exosomal carboxypeptidase econfers and CPE-shRNA loaded exosomes inhibit tumorigenesis

**Authors:** Sangeetha Hareendran, Bassam Albraidy, Xuyu Yang, Aiyi Liu, Anne Breggia, Clark C Chen, Y Peng Loh

**Affiliations:** ^1^Section on Cellular Neurobiology.; ^2^Biostatistics&Bioinformatics Branch, National Institute of Child Health and Human Development, NIH, Bethesda, MD, USA.; ^3^Maine Medical Center BioBank, Portland, ME, USA.; ^4^Department of Neurosurgery, University of Minnesota Medical School, MN, USA.

## Abstract

Carboxypeptidase E (CPE) and its splice variant CPE- N has been shown to promote cancer growth and metastasis in various cancer types. Exosomes carry biomolecules (proteins, DNA, mRNA and miRNA) unique to their cell of origin and deliver them to recipient target cells thereby mediating cell-cell communication. We have investigated whether exosomes from high metastatic HCCH (liver cancer) cells can confer growth and metastatic properties to HCCL (low metastatic). Exosomes were isolated from the supernatant culture media of cancer cells and a correlation was found between elevated CPE mRNA levels in exosomes from high *vs*. low metastatic cell lines across various cancer types. Content analysis of exosomes derived from HCC97H cells revealed CPE-WT mRNA and protein. We showed that exosomes released from HCC97H cells were able to enhance invasion of HCC cells with poor metastatic ability (HCC97L) in Matrigel invasion assay and proliferation in MTT assay. However, when CPE expression was suppressed in the HCC97H cells before exosome isolation, the exosomes had no effect on proliferation and invasion. These data demonstrate the ability of exosomes to confer metastasis in cancer cells and the role of exosomal CPE in driving the process. We then utilized the inherent property of exosomes to act as efficient delivery tools to carry a therapeutic agent such as shRNA. Previously it was shown that down-regulation of CPE expression by shRNA can reverse tumor growth and metastasis in an HCC mouse model. We therefore loaded CPE-shRNA into exosomes by infecting HEK293 (Human Embryonic Kidney) cells with adenovirus carrying CPE-shRNA-GFP. These modified exosomes were harvested from the cell medium, purified and then used to transfer CPE-shRNA to HCC97H cells. The exosomes taken up by the recipient cells resulted in significant reduction of CPE mRNA levels and decrease in colony formation of these cells. Thus, these studies demonstrate the ability of exosomal CPE to enhance invasion in low metastatic HCC cells and the potential to use shRNA loaded exosomes to target CPE as a therapeutic strategy to treat liver and other cancers. Finally, in a pilot study we measured CPE mRNA copy numbers in serum exosomes of patients with cancer (glioma, breast, ovarian, colon, cervical, pancreatic or prostate cancer) and healthy controls. Significantly elevated levels of CPE mRNA copy numbers were found in serum exosomes of cancer patients versus healthy controls, suggesting that exosomal CPE mRNA could potentially be used as a biomarker in an initial screen for diagnosing cancer.

# Other: 4. Bacterial vesicles: vehicles for Inter-Kingdom communication and modulators of plant immune response

**Authors:** Hannah M. McMillan, Sophia G. Zebell, Jean B. Ristaino, Xinnian Dong, Meta J. Kuehn

**Affiliations:** ^1^Department of Molecular Genetics and Microbiology, Duke University, Durham, NC, USA.; ^2^HHMI, Department of Biology, Duke University. Durham, NC, USA.; ^3^Department of Entomology and Plant Pathology, NC State University, Raleigh, NC, USA.; ^4^Department of Biochemistry, Duke University, Durham, NC, USA.

## Abstract

Bacterial outer membrane vesicles perform a variety of functions in bacterial survival and virulence. In mammalian systems, vesicles activate immune responses and have been exploited for use in vaccines. However, little work has focused on the role vesicles play in dry-land environments or in the context of plant-microbe interactions, and research has only just begun on their role as natural nanoscale vehicles. We show that vesicles from the plant pathogen Pseudomonas syringae pv tomato DC3000 and the plant beneficial Pseudomonas fluorescens activate plant immune responses that protect against future bacterial and oomycete challenge. Interestingly, our results also expose differences in vesicle packaging between pathogens and beneficials and in vesicles isolated from various environmental conditions that lead to different sensitivities to biochemical stressors. Importantly, these studies reveal unique differences in pathogen- versus beneficial-induced immune activation and support the use of vesicles as a tool to probe type III secretion system-independent immune responses. Uncovering the mechanisms through which pathogen-and beneficial-derived vesicles mediate plant immune responses and exploring their role as natural nanoparticle vehicles will reveal new targets for agricultural disease management and highlight novel roles for vesicles in overall ecosystem function.

# Other: 5. Staphylococcus aureus secretes immunomodulatory RNA via extracellular membrane vesicles

**Authors:** Blanca V. Rodriguez, Meta J. Kuehn

**Affiliations:** Department of Biochemistry, Duke University, Durham, NC, USA.

## Abstract

Bacterial-derived RNA can function as ligands for intracellular receptor activation and induce downstream signaling to modulate the host response to bacterial infection. The mechanisms underlying the secretion of immunomodulatory RNA by human pathogens, such as Staphylococcus aureus, and their delivery to intracellular host cell receptors are not well understood. Recently, extracellular membrane vesicle (MV) production has been proposed as a general secretion mechanism that could facilitate the delivery of functional bacterial nucleic acids into host cells. S. aureus produce membrane-bound, spherical, nano-sized, MVs packaged with a select array of bherefore, in the context of neurotrophic vioactive macromolecules and they have been shown to play important roles in bacterial virulence and in immune modulation through the transmission of biologic signals to host cells. The present study sought to examine the nature of the association between RNA and MVs produced by S. aureus. We also sought to analyze the immunostimulatory potential of MV- associated RNA and to evaluate receptor-mediated recognition of MV-associated RNA and DNA molecules by innate immune cells. Here we report that S. aureus produces MV-associated RNA molecules that are protected from nuclease degradation. MV-associated nucleic acids were transferred to cultured murine macrophages and induced significant Interferon-β mRNA expression largely through endosomal Toll-like receptor (TLR) signaling. Upon exposure to nuclease-treated MVs, TLR3-/- and TLR7-/- macrophages produced very little IFN-β mRNA. TLR3 recognizes dsRNA, which points to the possibility that S. aureus MVs are packaged with immunostimulatory dsRNA molecules. TLR7 has previously been found to recognize S. aureus tRNA, as well as ssRNA molecules. Altogether, these data indicate that endosomal nucleic acid receptors are activated in cultured mouse macrophages upon MV exposure, likely due to immunostimulatory properties of MV-associated nucleic acids. Our findings show for the first time an MV-mediated pathway by which *S. aureus*-derived immunomodulatory RNA molecules are delivered to host cells. How MV-associated nucleic acids are trafficked intracellularly and recognized by endosomal TLRs will be examined in future experiments.

# Other: 6. Separation of EVs from virions in coronavirus infections

**Authors:** James Erickson, Maria Cowen, Yuriy Kim, Anoop Pal, Heather Branscome, Archana Gupta, Fatah Kashanchi

**Affiliations:** ^1^Laboratory of Molecular Virology, School of Systems Biology, George Mason University, Manassas, VA, USA;; ^2^Izon Science (IZON), Medford, MA, USA.; ^3^American Type Culture Collection (ATCC), Manassas, VA, USA.; ^4^Systems Biosciences (SBI), Palo Alto, CA, USA.

## Abstract

Since the severe acute respiratory syndrome corona virus 2 (SARS-CoV-2) was declared a pandemic in mid-March of 2020 by the World Health Origination (WHO), laboratories around the world started research into diagnostics, therapeutics, and treatments^[1]^. In recent years, the importance of extracellular vesicles (EVs) in the pathogenesis of viral infections have been found in the cases of many viral pathogens including few DNA and RNA viruses including human T-cell leukemia virus-1 (HTLV-1) and human immunodeficiency virus-1 (HIV-1)^[2,3]^. EVs from HIV-1 infected cells on uninfected macrophages induces an increase in the proinflammatory cytokines^[3]^. While EVs from HTLV-1 infected cells on uninfected recipient cells promoted the localization and cellular contact by cells, this directly influences the pathogenesis of HTLV-1 as the virus mainly infects other cells by cell to cell contact^[2]^. Similar to retroviruses, coronaviruses are also positive strand RNA viruses, except they replicate in the cytoplasm and may regulate chromosomal DNA depending on the strain of virus. We have recently began working on beta- coronaviruses, including OC43 (BSL2 strain) and SARS-CoV-2 (BSL3 strain). Our initial experiments focus on isolation of EVs away from virions using either an iodixanol gradients or Izon sizing columns. We have successfully separated the two from one another mainly due to their density and potentially size differences. We found that EVs from multiple coronaviruses are not infectious and viral particles treated with UV irradiation are also not infectious. We also have found that coronavirus EVs caused T-cell death, which may corelate with lymphopenia observed in COVID patients. Along these lines coronavirus EVs can activate other viral genes (i.e., HIV-1 or HTLV-1) when these genes are integrated into the genome, further implying that these EVS regulate chromosomal gene expression. Finally, the mechanism(s) of how these EVs may cause such diverse effects on T-cells and other viral gene expression will be discussed.

## References

[1] Cucinotta D, Vanelli M (2020). WHO Declares COVID-19 a Pandemic.. Acta Biomed.

[2] Pinto DO, DeMarino C, Pleet ML (2019). HTLV-1 extracellular vesicles promote cell-to-cell contact.. Front Microbiol.

[3] Sampey GC, Saifuddin M, Schwab A (2016). Exosomes from HIV-1-infected cells stimulate production of pro-inflammatory cytokines through trans-activating response (TAR) RNA.. J Biol Chem.

# Other: 7. Use of stem cell extracellular vesicles as a holistic approach towards CNS repair

**Authors:** Heather Branscome, Siddhartha Paul, Dezhong Yin, Weidong Zhou, Lance A. Liotta, Fatah Kashanchi

**Affiliations:** ^1^Laboratory of Molecular Virology, School of Systems Biology, George Mason University, Manassas, VA, USA.; ^2^American Type Culture Collection (ATCC), Manassas, VA, USA.; ^3^ATCC Cell Systems, Gaithersburg, MD, USA.; ^4^Center for Applied Proteomics and Molecular Medicine, George Mason University, Manassas, VA, USA.

## Abstract

Neurological diseases and disorders are leading causes of death and disability worldwide. Many of these pathologies are associated with high levels of neuroinflammation and irreparable tissue damage. We have previously shown that extracellular vesicles (EVs) from infected cells contain viral by products (non-coding RNAs and proteins) and that these EVs can exert deleterious effects on recipient cells^[1-3]^. Therefore, in the context of neurotrophic viruses EVs may contribute to or perpetuate processes relating to neuroinflammation and neurodegeneration. Due to their multipotent properties, stem cells have broad applications for tissue repair; additionally, stem cells have been shown to possess both immunomodulatory and neuroprotective properties. In recent years it has been well-established that stem cell EVs play a critical role in the functionality associated with stem cells. The diverse biological cargo contained within these vesicles are proposed to mediate their effects and, to date, the reparative and regenerative effects of stem cell EVs have been demonstrated in a wide range of cell types. While a high potential for their therapeutic use exists, there is a gap of knowledge surrounding their characterization, mechanisms of action, and how they may regulate cells of the central nervous system (CNS). We have isolated and recovered high yields of EVs from large scale cultures of both induced pluripotent stem cells (iPSCs) and mesenchymal stem cells (MSCs) using tangential flow filtration. Our EV characterization includes both phenotypic (size, tetraspanin expression) and biochemical assays. EV functionality has also been assessed *in vitro* utilizing several cell-based assays related to cellular viability, migration, angiogenesis, and immunomodulation in both healthy and damaged recipient cells with relevance to the CNS. Our data suggests that EVs from different sources of stem cells display unique phenotypes, exhibit differential association with various cytokines, proteins, and long non-coding RNAs, and have the ability to significantly enhance processes that are critical for cellular repair^[4]^. Lastly, utilizing an iPSC-derived neurosphere model, we have observed a robust uptake of stem cell EVs and have found that these EVs are able to effectively penetrate these 3D structures. Collectively, these results highlight the “holistic” properties of stem cell EVs by demonstrating their ability to partially reverse or reduce damage in various cell types.

## References

[1] Sampey GC, Saifuddin M, Schwab A (2016). Exosomes from HIV-1-infected cells stimulate production of pro-inflammatory cytokines through trans-activating response (TAR) RNA.. J Biol Chem.

[2] Pleet ML, Erickson J, DeMarino C (2018). Ebola virus VP40 modulates cell cycle and biogenesis of extracellular vesicles.. J Infect Dis.

[3] Pinto DO, DeMarino C, Pleet ML (2019). HTLV-1 extracellular vesicles promote cell-to-cell contact.. Front Microbiol.

[4] Branscome H, Paul S, Khatkar P (2020). Stem cell extracellular vesicles and their potential to contribute to the repair of damaged CNS cells.. J Neuroimmune Pharmacol.

# Other: 8. Extracellular vesicles from HTLV-1 infected cells regulate viral spread and pathogenesis

**Authors:** Sarah Al Sharif, Daniel O. Pinto, Gifty Mensah, Maria Cowen, Pooja Khatkar, James Erickson, Heather Branscome, Catherine DeMarino, Weidong Zhou, Lance A. Liotta, Fatah Kashanchi

**Affiliations:** ^1^Laboratory of Molecular Virology, School of Systems Biology, George Mason University, Manassas, VA, USA.; ^2^Center for Applied Proteomics and Molecular Medicine, George Mason University, Manassas, VA, USA.

## Abstract

Human T-cell lymphotropic virus type 1 (HTLV-1) is the causative agent of adult T- cell leukemia/lymphoma (ATLL) and HTLV-1 associated myelopathy/tropical spastic paraparesis (HAM/TSP)^[1,2]^. Approximately 10 million worldwide are infected with HTLV-1, however, it is most likely that the true figure is greater than this number as many cases are not reported^[2]^. It is endemic in many areas such as southern Japan, the Caribbean, and parts of Africa^[2]^. In recent publications, we showed that HTLV-1 infected cells release Extracellular Vesicles (EVs) containing viral RNA and proteins (gp61/Tax/HBZ) and are not infectious^[3,4]^. However, they can enhance cell-to-cell contact of uninfected cells, eventually aid in spread of virus^[4]^. We separated distinct EV subpopulations from HTLV-1 infected cell supernatants by differential ultracentrifugation (DUC) into 2k, 10k, and 100k EVs based upon centrifugation speed. Proteome profiling of various HTLV-1 EV subpopulations showed that viral/host protein was highly abundant in 2k subpopulation, compared to other subpopulations. Western Blots revealed that p19 and Tax (i.e., viral proteins), as well as LC3 and p62 (i.e., autophagy proteins) were mainly present in the 2k and 10k subpopulation. Using an *in vitro* angiogenesis assay, 2k HTLV-1 EVs were primarily responsible for tubular deterioration. Different EV subpopulations were incubated with cells involved in neuroinflammation (i.e., astrocytes, macrophages, and neurons) where the highest level of IL-8 expression (involved in cell migration) was found in astrocytes and monocyte-derived macrophages. IL-6 (involved in inflammation) was only present in neurons treated with these EVs (i.e., 100k > 10k > 2k). HTLV-1 EVs were able to facilitate HTLV-1 viral spread in monocytic cell-derived dendritic cells via cell-cell contact. Finally, an increase in proviral DNA and RNA levels in humanized mice tissue (i.e., Blood, Lymph Node, and Spleen) were noticed following treatment with 2k and 10k HTLV-1 EVs, indicating the importance of these EVs in HTLV-1 spread. These findings indicate that different HTLV-1 EV subpopulations induce cytokine expression, tissue damage, and viral spread. These EV subpopulations could potentially contribute to the development of ATLL or HAM/TSP. Further mechanistic understanding of these EVs in HTLV-1 pathogenesis will be discussed for the development of preventative measures and treatment options for this devastating disease.

## References

[1] Gallo RC (2011). Research and discovery of the first human cancer virus, HTLV-1.. Best Pract Res Clin Haematol.

[2] Gessain A, Cassar O (2012). Epidemiological aspects and world distribution of HTLV-1 infection.. Front Microbiol.

[3] Jaworski E, Narayanan A, Van Duyne R (2014). Human T-lymphotropic virus type 1-infected cells secrete exosomes that contain tax protein.. J Biol Chem.

[4] Pinto DO, DeMarino C, Pleet ML (2019). HTLV-1 extracellular vesicles promote cell-to-cell contact.. Front Microbiol.

# Other: 9. Proteomics of cerebellar exosomal proteins: therapeutic and biomarker implications in spinocerebellar ataxia-1

**Authors:** Ribhav Mishra, Puneet Opal

**Affiliations:** Feinberg School of Medicine, Northwestern University, Chicago, IL, USA.

## Abstract

Exosomes are observed to carry proteins, RNA, and also some chromosomal DNA released by cells. These biomolecules are found to travel from one cell to another and thus acts as a messenger to communicate signals between different cells. In the nervous system, they are found to be critical for neuron-neuron, neuron-glia communications. The emerging roles of exosomes in different neurodegenerative pathologies like Alzheimer’s, Parkinson, and Prion suggests that exosomes can help in the spread of these toxic proteinaceous inclusions and hence they are a potential therapeutic target in such diseases. Exosomal biology in the diseases of Spinocerebellar ataxia-1 (SCA1) is not understood with no report present to define the role of exosomes in the SCA1 pathology. Since cerebellar cells are the primary cells affected in the SCA1, hence, we have characterized the exosomes isolated from the primary mixed culture of cerebellar cells using a variety of biochemical methods and biophysical techniques. Furthermore, we have identified the proteomic content of the exosomal lysate using the LC-MS/Mass Spectrometry and have found proteins that are enriched for the biological functions in the extracellular matrix, myelin sheath formation, axonal growth cone development. We will use this data to identify proteins that are necessary for the development and functions of the cerebellum and can derive a conclusion on the role of exosomes in SCA1. Exosomes also carry molecules that are relevant to disease-specific diagnosis and so they are also proposed as a biomarker tool in neurodegenerative pathologies of Alzheimer’s, Parkinson. However, their role as a biomarker candidate in SCA1 pathology is not defined and their use of a biomarker strategy is tested for the very first time by us. To validate exosomes as a biomarker in SCA1 pathology, we plan to isolate and characterize exosomes from the body fluids (blood and CSF) of WT^2Q/2Q^ and SCA1^154Q/2Q^ knock-in mouse models in different age groups of pre-symptomatic, early symptomatic, and late symptomatic. The exosomal proteins will be identified using the various proteomics-based approach and then compared between WT^2Q/2Q^ and SCA1^154Q/2Q^ knock-in mice and any alterations in the level of proteins will be studied further for use as a biomarker in SCA1 pathology. The results of the above work will also be tested as a biomarker candidate in SCA1 patients. The overall findings of our work will provide clues on the role of exosomes in the pathology of SCA1 and may well establish them as a biomarker in the pathology of SCA1.

# Other: 10. Advancing extracellular vesicle characterization with quantitative single molecule localization microscopy

**Authors:** Kathleen M Lennon, Adam L Maddox, Andras Saftics, Matthew S Brehove, Devin L Wakefield, Saumya Das, Kendall Van Keuren-Jensen, Gagandeep Singh, Tijana Jovanovic-Talisman

**Affiliations:** ^1^Beckman Research Institute, City of Hope, Duarte, CA, USA.; ^2^Cardiology Division, Massachusetts General Hospital, Harvard Medical School, Boston, MA, USA.; ^3^Neurogenomics Division, Translational Genomics Research Institute, Phoenix, AZ, USA.; ^4^City of Hope, Duarte, CA, USA.

## Abstract

Quantitative Single Molecule Localization Microscopy (qSMLM) can quantify individual biomolecules in cells with nanoscale precision. However, because of several technical hurdles, a similarly robust quantification is often difficult to achieve when the methodology is applied to extracellular vesicles (EVs). One significant challenge has been to rigorously isolate and characterize disease- specific and tissue-specific subpopulations of EVs. Our approach to probe EV subpopulations entails three main advancements: 1) more effective methods to label the EVs for SMLM imaging; 2) an optimized protocol for affinity isolation and imaging of EVs; and 3) advanced algorithms to robustly detect size and count the number of biomolecules within individual EVs. Briefly, size exclusion chromatography was first used to isolate EVs from either cultured cell media or patient plasma. Then, EV membranes were labeled with fluorescent reagents: dyes, lectins, or antibodies. The excess fluorescent molecules were removed by size separation using concentrating filters, core beads, or size exclusion columns. Finally, EVs enriched in specific receptor(s) were affinity isolated onto coverslips, imaged, and analyzed. Using this comprehensive approach, we have quantified EVs isolated from the plasma of patients who have pancreatic cancer. Specifically, we have determined the number of isolated EVs, their size, and the abundance of several biomarkers. Compared to healthy controls, patients with pancreatic cancer exhibited a distinct population of larger EVs enriched in epidermal growth factor receptor and carbohydrate antigen 19-9. Ultimately, when this approach is paired with traditional methods, it may shift the paradigm for comprehensively prehensively characterizing cancer-specific and organ-specific EVs.

# Other: 11. Hypocalcemic-induced exosome secretion drives differential metastatic progression of epithelial ovarian cancer

**Authors:** Amy H. Lee, Deepraj Ghosh, Nhat Quach, Michelle R. Dawson

**Affiliations:** ^1^School of Engineering, Center for Biomedical Engineering, Brown University, Providence, RI, USA.; ^2^Molecular Pharmacology, Physiology, and Biotechnology, Brown University, Providence, RI, USA.

## Abstract

**Introduction**: Serous epithelial ovarian cancer (EOC) is the most lethal gynecological malignancy and peritoneal fluid (ascites) build-up is common in late stage EOC. This ascites is an abundant source of cell-secreted exosomes. Gynecological conditions are often associated to decreased blood serum calcium concentrations and elevated exosome production. Exosomes are continuously secreted through the endosomal pathway; however, their release can be triggered by various physiologic stimuli, leading to exosome heterogeneity. We have demonstrated that mimicking hypocalcemia conditions by chelating extracellular calcium releases a unique population of exosomes from an EOC cancer cell line. We hypothesize that naturally secreted exosome (SEC-exosomes) *vs*. chelation-induced exosome (CI-exosomes) treatment will lead to differential biophysical and molecular cellular responses to mediate more invasive tumor microenvironment (TME).

**Methods**: OVCAR-3 EOC cells were cultured in exosome-free media for 48 hours. SEC-exosomes were collected from media and harvested using standard ultracentrifugation methods. OVCAR-3 were washed and treated with EDTA to harvest CI-exosomes. CI-exosomes were isolated using ultracentrifugation. Exosome diameters and ubiquitous surface protein markers for CI- and SEC- exosomes were validated using dynamic light scattering and immunoblots, respectively. Patient-derived EOC fibroblasts were treated with either exosome population. High-throughput physical and molecular assays - single-cell migration, immunocytochemistry, adhesion assays, and miRNA microarrays - were used to examine unique differences between populations and their interactions with fibroblasts.

**Results**: Microarray data showed unique miRNA profiles in SEC- *vs*. CI- exosomes, suggesting heterogeneity in cell-secreted exosomes. Specifically, there were 1,019 differentially expressed miRNAs between either exosome population. Highly regulated miRNAs were linked to mechanosensitive pathways that impact cell motility and cytoskeletal organization. Both populations altered actin fiber and focal adhesion protein organization to affect fibroblast morphologies. Exosome populations further increased random and directional fibroblast migration; however, fibroblast adhesion strength was only increased with CI-exosome treatment. For co-culture OVCAR-3 and fibroblast studies, CI-exosomes promoted adhesion and spreading of OVCAR-3 cells, while SEC-exosomes led to dramatic elongation in a small percentage of OVCAR-3 cells.

**Discussion/Conclusions**: We showed that decreased extracellular calcium levels, which mirror various gynecological conditions, led to the release of a unique exosome population (CI-exosomes) from a single cancer cell line. This CI-exosome population contained unique miRNA content and led to different molecular/physical fibroblast phenotypes compared to SEC-exosomes. This highlights that tumor cells can secrete multiple exosome populations to mediate cancer progression.

# Other: 12. Orchestration of human macrophage NLRP3 inflammasome activation by Staphylococcus aureus extracellular vesicles

**Authors:** Jean C. Lee, Xiaogang Wang

**Affiliations:** Division of Infectious Diseases, Department of Medicine, Brigham and Women’s Hospital and Harvard Medical School, Boston, MA, USA.

## Abstract

*Staphylococcus aureus* (*S. aureus*) is an opportunistic pathogen that causes a variety of diseases, including bacteremia, endocarditis, skin and soft tissue infections, and food poisoning. The bacterium produces a myriad of virulence factors, including toxins, enzymes, surface proteins, and multiple glycopolymers, many of which are components of staphylococcal extracellular vesicles (EVs). We purified EVs from a community-associated methicillin-resistant *S. aureus* strain and showed that EV-associated pore-forming toxins, particularly alpha hemolysin and members of the leukocidin family, lysed a variety of cell types. Staphylococcal alpha-type phenol-soluble modulins promoted EV biogenesis by disrupting the cytoplasmic membrane, whereas peptidoglycan crosslinking and autolysin activity modulated EV production by altering the permeability of the cell wall. Purified *S. aureus* EVs were internalized into human macrophages in vitro, and this process was blocked by inhibition of the dynamin-dependent endocytic pathway. EVs triggered NLRP3 inflammasome activation, resulting in the cellular release of IL-1β and IL-18 and induction of pyroptosis. Consistent with this result, a dose-dependent cytokine response was detected in the extracellular fluids of mice challenged intraperitoneally with *S. aureus* EVs. Pore-forming toxins associated with EVs were critical for NLRP3-dependent caspase-1 activation of human macrophages, but not for TLR2 signaling. In contrast, EV-associated lipoproteins not only mediated TLR2 signaling to initiate the priming step of NLRP3 activation but also modulated the toxin content and the biogenesis of EVs, resulting in alterations in IL-1β, IL-18, and caspase-1 activity. Our study describes mechanisms by which *S. aureus* EVs induces inflammasome activation and reveals an unexpected role of staphylococcal lipoproteins in EV biogenesis. S. aureus EVs may serve as a novel secretory pathway to transport protected cargo to host cells during infection to modulate cellular functions.

# Other: 13. Beta-glucan stimulated neutrophils secrete IL-1α through exosomes

**Authors:** Bridget Ratitong, Michaela E. Marshall, Eric Pearlman

**Affiliations:** Department of Physiology and Biophysics, University of California, Irvine, CA, USA.

## Abstract

Interleukin-1α (IL-1α) and IL-1β are leaderless pro-inflammatory members of the IL-1 family of cytokines that are secreted via unconventional, ER and Golgi-independent pathways. Secretion of processed, bioactive IL-1β by macrophages is mediated by Gasdermin D (GSDMD); in contrast, little is known about the mechanism of IL-1α release by myeloid cells. Here, we demonstrate that IL-1α production by macrophages is completely dependent on Gasdermin D. We found that neutrophils produce IL-1α following inflammation induced by Aspergillus fumigatus spores that express cell surface β -glucan, and show that in contrast to macrophages, IL-1α secretion by β-glucan stimulated neutrophils occurs independently of GSDMD. Instead, we found intracellular IL-1α co-localized with extracellular vesicle (EV) markers CD63, CD9, and CD81, and that IL-1α is encapsulated in EVs isolated from β-glucan stimulated neutrophils. Preincubation of neutrophils with GW4869 inhibited exosome release and significantly reduced IL-1α levels in cell-free supernatant. Further, we found that exosomal IL-1α can signal through its receptor, IL-1R1, and EVs isolated from neutrophils, and can activate macrophages as measured by proinflammatory cytokines IL-6 and TNFα release. Collectively, these findings demonstrate that the mechanism for IL-1α secretion in neutrophils is distinct from macrophages and identify a role for neutrophil extracellular vesicles, specifically exosomes, in this process.

# 14. Hypoxia-responsive bovine milk exosomes as targeted carrier for chemotherapeutics

**Authors:** Jessica Pullan, Li Feng, Kaitlin Dailey, Sangeeta Bhallamudi, James Froberg, Lina Alhalhooly, Amanda Brooks, Sathish Venkatachalem, Yongki Choi, Sanku Mallik

**Affiliations:** ^1^Department of Pharmaceutical Sciences, North Dakota State University, Fargo, ND, USA.; ^2^Department of Physics, North Dakota State University, Fargo, ND, USA.; ^3^Department of Research and Scholarly Activity, Rocky Vista University, Ivins, UT, USA.

## Abstract

Exosomes are secreted nanosized extracellular vesicles with innate transportation ability making them ideal candidates for drug delivery. The inherent cellular cargo of an exosome can negatively impact its ability to carry and deliver cargo safely. Bovine milk exosomes have not shown the same effect as other sources in throughout the literature and therefore are a safe and effective source of exosomes. In order to increase targeting and predictable control drug release of bovine exosomes, a neuropilin receptor agonist peptide (iRGD) and a hypoxia-responsive lipid have been incorporated into the lipid bilayer of bovine milk exosomes. Hence, peptide targeted, hypoxia-responsive bovine milk exosomes (iHRX) encapsulating the anticancer drug doxorubicin will decrease cell survival of triple-negative breast cancer cell population. Doxorubicin has been encapsulated using electroporation and shows encapsulation efficiencies of above 50%. Transmission electron microscopy, atomic force microscopy, and dynamic light scattering have demonstrated the stability of modified exosomes in normoxic conditions and exosome fragmentation under concentrations greater than 5 mM glutathione, which directly corresponds to tumor oxygen levels. Additional studies to confirm the presence of iRGD has been performed indicating strong adhesion to the avb3 integrin most abundantly found in tumors. Future studies will include testing of iHRX’s toxicity and cellular internalization in monolayer and three-dimensional cultures prior to *in vivo* studies.

# Other: 15. Liver tumor-derived exosomes (small extracellular vesicles) induce macrophage polarity

**Authors:** Mackenzie J. Burroughs, Gina T. Bardi, Joshua L. Hood

**Affiliations:** Department of Pharmacology and Toxicology, University of Louisville, Louisville, KY, USA.; James Graham Brown Cancer Center, University of Louisville, Louisville, KY, USA.; Hepatobiology and Toxicology COBRE, University of Louisville, Louisville, KY, USA.

## Abstract

Our recent data demonstrate that nanoscale tumor-derived small extracellular vesicles (sEVs), otherwise known as exosomes, induce macrophage (Mφ) functions. Tumor-associated M1 (proinflammatory) or M2 (immunosuppressive) Mφs are pivotal in the progression of inflammation-related cancers such as hepatocellular carcinoma. However, the ability of liver tumor-derived sEVs to influence Mφ- related pro-tumor processes is largely unexplored. Using human HepG2 liver tumor and THP-1 Mφ models, we investigated the hypothesis that HepG2-sEVs induce Mφ polarity, indicative of pro-tumor inflammatory processes. Preliminary ELISA results revealed a dose dependent increase in production of the proinflammatory M1 cytokine TNF-α and immunosuppressive M2 cytokine IL-10 at a treatment concentration of 0.05 μg sEV protein/μL. Subsequent ELISA experiments demonstrated batch to batch variations in the induction of IL-10 by HepG2-sEVs at the 0.05 μg sEV protein/μL dose. In contrast, normal plasma (np) control sEVs did not increase IL-10 production. TNF-α production was significantly induced by all np-SEV and HepG2-sEV lots and batches tested. Use of qRT-PCR showed that both np control and HepG2 sEVs favored induction of M1 polarization factors with HepG2’s also trending toward induction of a few mixed M1/M2 and M2 markers. Overall, HepG2-sEVs induced Mφ polarity, indicative of pro-tumor inflammatory processes. Future investigations will explore these processes and derivative therapeutic strategies.

**Grant Support**: University of Louisville Cancer Education Program (R25-CA134283), The Elsa U. Pardee Foundation (PI J.L. Hood), University of Louisville Department of Pharmacology and Toxicology Faculty Start-up funds to J.L. Hood, and UofL Hepatobiology and Toxicology COBRE NIGMS NIH P20GM113226.

# Other: 16. Small RNA-binding molecules inhibit HIV-1 transcription in CNS latent reservoirs

**Authors:** Pooja Khatkar, Maria Cowen, Catherine DeMarino, Fardokht A Abulwerdi, Lance A Liotta, Stuart F.J Le Grice, Fatah Kashanchi

**Affiliations:** ^1^Laboratory of Molecular Virology, George Mason University, Manassas, VA, USA.; ^2^Infections of the Nervous System, NINDS, Bethesda, MD, USA.; ^3^Basic Research Laboratory National Cancer Institute, Frederick, MD, USA.; ^4^Center for Applied Proteomics and Molecular Medicine, George Mason University, Manassas, VA, USA.

## Abstract

Despite the emergence of combinational antiretroviral therapy (cART), 50% of all HIV-1 infected patients demonstrate some degree of HIV-1 associated neurocognitive disorder ranging from asymptomatic neurocognitive impairment to mild neurocognitive disorder to severe HIV-associated dementia. Although cART is highly effective in suppressing viremia, the lack of an FDA approved viral transcription inhibitor allows for persistent non-processive transcription which results in the production of short non-coding RNAs (TAR and TAR-gag)^[1,2]^. Our lab and others have previously shown that these RNAs are released from the cell in extracellular vesicles (EVs), specifically exosomes, and can induce production of proinflammatory cytokines in recipient myeloid cells^[3,4]^; a factor that may contribute to the neuroinflammation observed in HIV-1 infected individuals with HIV associated neurocognitive disorder (HAND). Here, we have screened a panel of small molecules that have been previously found to noncanonically bind HIV-1 Trans Activation Response (TAR) element, a 5’ RNA element that is required for activated viral transcription^[5]^. We have identified 5 candidates that effectively inhibit viral transcription in myeloid and T-cells without toxicity. The mechanism of inhibition may be directly linked to Tat-activated transcription and epigenetics modification. These data also suggest a direct link and communication between Nuclear Transcription and EV cargo biogenesis. Collectively, our results suggest that the use of small RNA-binding molecules to inhibit viral transcription could potentially be used to complement existing cART drugs to address the current therapeutic gap in current regimens.

## References

[1] Barclay RA, Schwab A, DeMarino C (2017). Exosomes from uninfected cells activate transcription of latent HIV-1.. J Biol Chem.

[2] Akpamagbo YA, DeMarino C, Pleet ML (2017). HIV-1 transcription inhibitors increase the synthesis of viral non-coding RNA that contribute to latency.. Curr Pharm Des.

[3] Sampey GC, Saifuddin M, Schwab A (2016). Exosomes from HIV-1-infected cells stimulate production of pro-inflammatory cytokines through trans-activating response (TAR) RNA.. J Biol Chem.

[4] DeMarino C, Pleet ML, Cowen M (2018). Publisher correction: antiretroviral drugs alter the content of extracellular vesicles from HIV-1-infected cells.. Sci Rep.

[5] Abulwerdi FA, Shortridge MD, Sztuba-Solinska J (2016). Development of small molecules with a noncanonical binding mode to HIV-1 trans activation response (TAR) RNA.. J Med Chem.

# Other: 17. Extracellular vesicles and microRNAs in maternal milk are important for growth and gut health during weaning in murine pups

**Authors:** Janos Zempleni, Mahrou Sadri, Fang Zhou

**Affiliations:** Department of Nutrition and Health Sciences, University of Nebraska-Lincoln, Lincoln, NE, USA.

## Abstract

**Background**: Human milk contains approximately 2.2×10(exp)11 exosome-size EVs per mL, which harbor more than 200 microRNAs. Mammals absorb EVs and microRNAs from milk, and microRNAs regulate approximately 60% of human genes. Studies in transgenic mice suggest that EVs in maternal milk accumulate primarily in the liver, brain and gastrointestinal (GI) mucosa in neonate pups.

**Objective**: Assess whether maternal EVs and microRNAs promote postnatal growth and GI health in neonate mice.

**Methods**: WT pups were fostered to homozygous Tsg101 KO dams (impaired exosome biogenesis), heterozygous Dicer KO dams (loss of microRNA biogenesis) or WT dams (control) from synchronized pregnancies (4 pups/dam). We assessed milk EVs and microRNAs, gut development, barrier function, mRNA expression profile in the jejunum, postnatal weight gain, and milk quality and intake. Statistics: unpaired *t*-test (Tsg101/Dicer *vs*. control); *P* < 0.05.

**Results**: KO of TSG101 and Dicer caused an 80% and 60% decrease of EVs and microRNAs, respectively, in milk. The loss of milk EVs and microRNAs led to an up to 20% shorter length of the gut, 20% decrease of villi height and 15% crypt depth, 50% increase in leakiness of the gut (appearance of FITC-dextran in blood), and a 50% loss of postnatal weight gain in pups. Approximately 400 mRNAs were differentially expressed in the jejunums of pups fostered to TSG101 KO dams or WT dams. Nutritional quality of milk and milk intake were not study confounders.

**Conclusions**: Mothers communicate with their offspring through EVs and microRNAs in milk, and the maternal message plays a role in optimal growth and gut health in neonate mice.

**Funding**: NIFA/USDA 2016-67001-25301 and 2020-67017-30834, NIH P20GM104320, USDA Hatch and W-40022 and Gates Foundation OPP1200494. J.Z. is a consultant for PureTech Health, Inc.

# Other: 18. High-capacity membranes for simple, rapid extracellular vesicle isolation with high yield and purity

**Authors:** Yi Zhao, Brenda Huang, Boris Levitan, Michael Haugwitz, Andrew A. Farmer

**Affiliations:** Takara Bio USA, Inc., Mountain View, CA 94043, USA.

## Abstract

**Introduction**: Despite their small size, extracellular vesicles (EVs), such as exosomes, play important roles in normal physiological processes and diseases. A critical bottleneck in EV research is the isolation of the vesicles, which has historically been accomplished via ultracentrifugation (UC). However, UC is time consuming, is not scalable, requires specialized equipment, may damage vesicles during the high- speed spins, can pull down non-EV proteins and nucleic acids, and suffers from low yield. More recently, precipitation solutions have been utilized to simplify EV isolation protocols, but these techniques are often inconsistent, provide low yields, and reduce purity. Thus, there is a strong need for a rapid EV isolation method that does not compromise purity or yield.

In order to overcome these shortcomings, we developed a new method of purifying EVs using a membrane-column-based approach. This method comprises a novel membrane conjugated to a lectin-based compound that selectively binds EVs. The membrane is chemically modified to have increased surface area, allowing higher binding capacity and yield, while also providing a highly pure and concentrated EV preparation. Additionally, the membrane is assembled into benchtop-centrifuge- compatible spin columns, which can be used to isolate EVs in under 30 minutes, improving on lengthy UC protocols. These kits provide a new and reliable method to rapidly enrich EVs from biological fluids for downstream analyses.

**Results**: Capturem-isolated EVs exhibited a more uniform and smaller particle size distribution, with an average particle size of 81 nm and a D90 value of 110 nm. Conversely, UC-isolated EVs were larger, with an average particle size of 135 nm and a D90 value of 203 nm. Additionally, the particle sizes isolated from UC were more variable than those from Capturem isolation. EVs isolated from plasma using the Capturem kit or UC were labeled with fluorescent dye and subjected to fNTA analysis. The results showed that UC- isolated EVs were highly contaminated with other particles and only 20% exhibited EV-specific labeling. In contrast, Capturem-isolated EVs demonstrated more than 4X this enrichment, with over 84% EV-specific labeling. Thus, Capturem columns consistently provide a pure, intact, and concentrated EV population in less than 30 minutes.

**Conclusions**: Research and clinical studies could greatly benefit from technology that provides rapid and simple isolation of EVs from various biofluids-especially as it concerns methods that generate pure, concentrated EV samples with enough yield for subsequent proteomic, genomic, and transcriptomic analyses. We have designed the Capturem™ Extracellular Vesicle Isolation Kit to meet these high standards, enabling researchers to consistently obtain pure EVs. These tools have the potential to enable faster drug discovery and better diagnostics for a variety of diseases.

# Other: 19. Isolation and characterization of extracellular vesicles in saliva of children with asthma

**Authors:** Nicole Comfort, Tessa Bloomquist, Amparito Cunningham, Marissa Hauptman, Wanda Phipatanakul, Andrea Baccarelli

**Affiliations:** Department of Environmental Health Sciences, Mailman School of Public Health, Columbia University, New York, NY, USA.; Harvard Medical School, Boston, MA, USA.; Division of Allergy and Immunology, Boston Children’s Hospital, Boston, MA, USA.

## Abstract

Asthma is the most common chronic disorder in children and is a heterogeneous disease, making identification of objective biomarkers of its subtypes and their underlying pathology a research priority. Identifying biomarkers that can profile clinical subtypes early in the course of asthma is critical in applying tailored therapy, yet the clinical utility of current biomarkers is very limited because they are either invasive or non-specific and unable to identify clinical subtypes. Non- invasive methods to study and monitor childhood asthma severity and distinguish pathophysiological subgroups is a critical research gap. Extracellular vesicles (EVs) and their bioactive cargo are attractive candidates for biomarkers of asthma endotypes.

Saliva, regarded as the “mirror of the body”, harbors constituents that provide sources for monitoring of health and disease states. Thus, we hypothesize that saliva EVs and their cargo may serve as a novel biomarker for asthma subtypes. Here, we first confirm the presence of EVs in saliva of children with asthma and characterize the EV population isolated from saliva supernatant using a high- throughput EV isolation method that can be scaled to large epidemiological studies of childhood asthma such as the School Inner City Asthma Intervention Study (SICAS-2).

**Objective**: To characterize the population of EVs isolated from saliva supernatant of children with asthma. Methods: EVs were isolated from saliva supernatant of 209 SICAS-2 study participants using ExoQuick-TC (System Biosciences) following a modified protocol. Pelleted EVs were re-suspended in filtered 1X dPBS. 5 μL of re-suspended EVs were aliquoted from each sample and combined to create a pooled sample for downstream analyses. EVs were visualized by morphological analyses (TEM). Capillary immunoassays confirmed the presence of EV-associated proteins. Microfluidic Resistive Pulse Sensing (MRPS) and Nanoparticle Tracking Analysis (NTA) were performed to determine EV concentration and size distribution. The concentration and size distribution of specific tetraspanin subpopulations (CD9, CD63, CD81) was determined using the ExoView R100 (NanoView Biosciences).

**Results**: Rounded, membrane-bound structures (25-350 nm) identified by TEM were positive for CD9, CD63, CD81, ANXA5, and ICAM-1 with limited cellular contamination (negative for CANX). MRPS detected a broad distribution of particle sizes with a significant population at 800 nm (concentration of peak population: 5.9 × 10^6^ particles/mL, 0.01× stock). Further analysis of small (< 200 nm) EVs with fluorescent NTA revealed a bi-modal distribution of EVs at 90 nm and 150 nm. 28% of EVs were positive for CD9, 35% were double positive for CD9 and CD63, 25% expressed CD9 and CD81, and 12% expressed all three tetraspanins.

**Conclusions**: Our findings characterize saliva EVs from children with asthma and show that saliva EVs can be isolated in a high-throughput method, opening a new avenue for asthma epidemiology studies.

# Other: 20. Immunoregulatory properties of cancer stem cell derived extracellular vesicles

**Authors:** Jordan Pavlic, Joehleen Archard, Jan Nolta, Johnathon D. Anderson

**Affiliations:** Stem Cell Program &Institute for Regenerative Cures University of California Davis Health Systems, University of California Davis, Sacramento, CA, USA.; Department of Biological Sciences, California State University, Sacramento, CA, USA.; Department of Otolaryngology University of California Davis Health Systems, California Institute for Regenerative Medicine, University of California Davis, Sacramento, CA, USA.

## Abstract

Head and neck squamous cell carcinoma (HNSCC) is the 6th most common malignancy globally, and the five year survival rate for stage III and IV patients is only 50%. HNSCC patients who relapse have a mean survival rate of less than one year. As such, understanding the mechanism of local recurrences and their immunoevasive properties is imperative to improving disease outcomes for stage III and IV HNSCC patients. Cancer stem cells (CSCs) are a subpopulation of cells within a tumor that possess self-renewing properties that have been linked to tumor initiation, neoplastic maintenance, and recurrence post treatment. Residual CSCs not eliminated by surgical resection, or chemoradiation, are thought to induce recurrence in many patients. A key feature of CSCs is their immunoregulatory properties that allow them to evade immunosurveillance and elimination. Several studies have established that CSCs secrete canonical secretory proteins with immunoregulatory properties, however, it remains unclear what role, if any, extracellular vesicles play in CSCs immunomodulatory properties. Using a co-culture system and multichromatic flow cytometry analysis we have evaluated CSCs and their derived EVs ability to regulate the polarization of the monocyte cell line, THP-1 macrophages towards an M2-like immunosuppressive phenotype. We have also analyzed the expression patterns of inhibitory immune checkpoint receptors and glycan factors in CSCs and CSC-EVs. Our preliminary data suggests that CSCs and CSC-EVs express numerous immune checkpoint proteins and associated glycans such as sialic acid residues. Our data also suggests that CSC-EVs induce M2-like polarization of macrophages.

# Other: 21. Extracellular vesicles in diabetes mellitus carry inflammatory cargo that affects cellular behavior

**Authors:** Nicole Noren Hooten, Sharon F. Wu, David W. Freeman, Nicolle A. Mode, Alan B. Zonderman, Michele K. Evans

**Affiliations:** ^1^Laboratory of Epidemiology and Population Science, National Institute on Aging, National Institutes of Health, Baltimore, MD, USA.; ^2^Kansas City University of Medicine and Biosciences, Kansas City, MO, USA.; ^3^University of Utah School of Medicine, Salt Lake City, UT, USA.

## Abstract

Type 2 diabetes mellitus is a global health problem evidenced by its rising prevalence and incidence worldwide. This chronic metabolic disease causes multiple end organ complications including inflammation-related atherosclerotic peripheral vascular disease, which results in devastating morbidity and mortality. Previously, we reported that individuals with diabetes mellitus have higher levels of circulating extracellular vesicles (EVs). EVs from diabetic individuals are more readily internalized by monocytes and induce inflammatory signals in these cells. To further elucidate the molecular cargo that may elicit these responses in cells, we quantified inflammatory protein levels in plasma-derived EVs from a longitudinal cohort of euglycemic and diabetic individuals. We report many significant associations between EV inflammatory protein levels and diabetes status. Most importantly, we found that vascular endothelial growth factor A (VEGF-A) was significantly associated with diabetes status in our longitudinal cohort. The plasma levels of this angiogenic factor were higher in EVs from individuals with diabetes compared to euglycemic individuals. Furthermore, EV levels of VEGF-A were significantly associated with homeostatic model assessments of insulin resistance (HOMA-IR) and β-cell function (HOMA-B), which are mathematical models that measure diabetes severity. To examine whether EVs with different inflammatory cargo can affect target cell behavior, we performed *in vitro* cell biological assays to assess the functional effects of these EVs on endothelial cells. We found that EVs from diabetic individuals induced actin cytoskeletal rearrangements including cell lamellipodia formation and increased cellular migration when compared to EVs from euglycemic individuals. Here we demonstrate that EV inflammatory protein profiles differ by diabetes status. Hence, our data suggest that EVs may play important roles in the end organ complications of diabetes mellitus, such as peripheral vascular disease.

# Other: 22. IMP1 modulates extracellular vesicle production in the GI tract through regulation of endosome and autophagy pathways

**Authors:** Sarah F. Andres, Madeline Kuhn, Ranjan Preet, Aurora Blucher, John Favate, Sukanya Das, Shun Liang, Louis Parham, Priya Chatterji, Wei Zhang, Jiegang Yang, Kathryn E. Hamilton, Premal Shah, Gordon Mills, Dan A. Dixon, Wei Guo, Anil K. Rustgi

**Affiliations:** School of Medicine, Oregon Health and Science University, Portland, OR, USA.; Department of Molecular Biosciences, University of Kansas, Lawrence, KS, USA.; Department of Genetics, Rutgers University, Newark, NJ, USA.; Divisions of Gastroenterology, Children’s Hospital of Philadelphia and University of Pennsylvania, Philadelphia, PA, USA.; Columbia University Irving Medical Center New York, NY, USA.

## Abstract

**Background**: RNA binding proteins regulate protein expression through coordinated binding to a series of related transcripts referred to as an RNA operon. Previous work demonstrates context and tissue specific roles for the RNA binding protein insulin-like growth factor 2 mRNA binding protein 1 (IMP1) in the context of cancer, especially colorectal cancer (CRC). Recent studies have linked IMP1 to extracellular vesicles and demonstrated that Imp1 is a potential regulator of autophagy during intestinal repair in response to damage. The endosomal, extracellular vesicle, and autophagy pathways are interrelated; however, a direct role for IMP1 in coordinating these pathways in the GI tract has not been explored. Hypothesis: IMP1 alters vesicle production and secretion by altering expression of vesicle pathway proteins in colon cancer and the non-transformed intestinal epithelium.

**Methods**: Ribosomal profiling and RNA sequencing were used to compare transcript and translational efficiency profiles between IMP1 null and IMP1- overexpressing CRC cell lines. Extracellular vesicles were assessed by nanoparticle tracking analysis, western blot, and electron microscopy in CRC cells and non- transformed, mouse intestinal enteroids. Effects of IMP1 on endosome, vesicle, and autophagy pathways were assessed by electron microscopy, immunofluorescence, and western blot in cell lines and enteroids.

**Results**: A number of extracellular vesicle and exosome-related pathways were the most significant differentially regulated pathways by gene ontogeny analysis of RNA sequencing data in IMP1 null *vs*. IMP1-overpressing CRC cells (*P* = 1.78×10^-10^, 2.43×10^-10^). Over 42% of the changed transcripts are associated with a vesicle pathway and differentially regulated by IMP1 expression. We found that IMP1 increases extracellular vesicle secretion in HT-29 (4483 +/- 403 *vs*. 1934+/- 414, *P* = 0.006) and SW480 (3604+/- 399 *vs*. 2293+/- 464, *P* = 0.049) CRC cells *vs*. null controls. IMP1 modulates translational efficiency or protein levels of early endosome proteins, including: EEA1, SNX15, PLA2G4B, and F8A2. IMP1 decreases expression of autophagy proteins LAMP1 and LC3-II. By contrast, Imp1 overexpression in non-transformed enteroids does not enhance vesicle secretion, but may alter expression of endocytic proteins.

**Conclusions**: Our novel findings suggest that IMP1 plays an important role in vesicle production and secretion in the context of colon cancer through increased early endosomal pathway activity and reduced autophagy-mediated degradation. Importantly, the effects of IMP1 expression differ between non-transformed and cancer cells. Our findings have implications for the development of novel early detection and therapeutic approaches in CRC where IMP1 is overexpressed.

**Funding**: AGA Research Scholar Award, Prevent Cancer Foundation Fellowship Award, Lustgarten Family Colon Cancer Research Fund, NIH F32DK107052, T32CA1152999, R01DK056645, P30CA13696, R01CA243445.

# Other: 23. Simple workflow for isolation and Western blot detection of MISEV-recommended EV protein-markers

**Authors:** Lisa Meyer, Daniel Enderle, Carsten Lück, Anne Bickel, Yasef Khan, Chris Heger, Martin Schlumpberger, Markus Sprenger-Haussels, Johan Skog, Mikkel Noerholm

**Affiliations:** ^1^Exosome Diagnostics GmbH, Martinsried, Germany.; ^2^Bio-Techne, Analytical Solutions Division, Abingdon, IN, USA.; ^3^Bio-Techne, Analytical Solutions Division, San Jose, CA, USA.; ^4^QIAGEN GmbH, Hilden, Germany.; ^5^Exosome Diagnostics, Inc., Waltham, MA, USA.

## Abstract

**Introduction**: Characterization of isolated extracellular vesicles (EV) is often challenging, due to the limited amount of material and the variety in approaches for experimental EV preparations. The “Minimal Information for Studies of Extracellular Vesicles” (MISEV) guidelines advice to characterize isolated EVs by their protein composition. Nevertheless, following these recommendations can result in a significant effort due to work-intensive and variable EV isolation procedures and time-consuming standard western blot protocols. Here we present a complete workflow for isolating intact EVs via membrane-affinity columns and detection of MISEV-recommended protein markers using Simple Western Blotting.

**Methods:** Intact EVs were isolated using a bind-wash elute procedure from 0.25-8 mL pre-filtered plasma or 10 mL of pre-filtered urine (exoEasy Kit, QIAGEN). EVs were eluted in 250 µL elution buffer for analysis and compared to the EV-depleted fraction in the column flow-through and to the neat biofluid sample. Multiple EV and non-EV protein markers were analyzed by subjecting 4 μL sample directly to Simple Western system, which entails automated capillary electrophoresis-based protein separation and chemiluminescence-immunodetection (Jess platform, ProteinSimple, Bio-Techne).

**Results:** A total of ten primary antibodies were identified and optimized for the workflow, including multiple targets in each of the three categories suggested by the MISEV-guidelines. Input volume titrations were used to define the dynamic range for the complete workflow and replicate isolations were used to demonstrate its repeatability. EV markers are generally low abundant in urine and plasma but were readily detected after enrichment of EVs by isolation. The EV fractions showed a strong enrichment of EV-proteins, whereas non-EV proteins were significantly reduced - and increasing the number of wash steps in the EV isolation lead to a substantially improved signal-to-noise ratio.

**Conclusions:** We developed a simple, robust and quantitative workflow for isolating and analyzing EV proteins. EVs obtained by membrane-affinity spin columns were enriched in the relevant EV proteins and depleted for non-EV proteins, establishing a method for easy compliance with official MISEV guidelines.

# Other: 24. Nanomechanical fingerprinting of single extracellular vesicles

**Authors:** Shivani Sharma

**Affiliations:** Department of Pathology & Laboratory Medicine, David Geffen School of Medicine, University of California Los Angeles, Los Angeles, USA.; Jonsson Comprehensive Cancer Center, University of California Los Angeles, Los Angeles, CA, USA.

## Abstract

As potential a class of novel diagnostics and therapeutics, the physio-chemical characterization as well as the biomolecular composition of EVs are widely investigated. However, there is emerging evidence suggesting that biomechanical analysis of lipid-bilayer membrane-bound single EVs may provide key insights into their biological structure, biomarker functions, and potential therapeutic functions. Using multi-parametric AFM imaging and force spectroscopy we compared the structure-mechanical properties (including Young’s modulus, stiffness, deformability, and adhesion maps) of invasive and noninvasive breast cancer EVs at nanoscale resolution. Our findings reveal that secreted EVs reflect the biomechanical signatures of parent cancer cells that vary in invasive potential. Irrespective of the EV isolation method employed, single EVs derived from non- invasive (biomechanically stiffer) cancer cells were also significantly biomechanically distinct compared to EVs derived from invasive (biomechanically soft) breast cancer cells. In particular, we propose multi-parametric AFM structure- mechanical analysis augmented with machine learning capabilities to further advance label-free, orthogonal biophysical understanding of EVs beyond biomolecular or particle size characterization and analysis, with significant implications for research and clinical use of EVs.

# Other: 25. Development of non-invasive clinically applicable *in vivo* tracking of extracellular vesicles using magnetic resonance imaging

**Authors:** Johnny Akers, Paola Aguiari, Hasmik Soloyan, Seda Mkhitaryan, Gevorg Karapetyan, Laura Perin, Mya Thu, Sargis Sedrakyan

**Affiliations:** ^1^VisiCELL Medical Inc, San Diego, CA, USA.; ^2^Children’s Hospital Los Angeles/University of Southern California, Los Angeles, CA, USA.

## Abstract

As researchers continue to explore the therapeutic potentials of extracellular vesicles (EVs) for the treatment of many diseases, there is a growing unmet need for real-time *in vivo* monitoring of these therapeutic EVs after they are injected into a subject to understand their safety, targeting, and effectiveness. While current optical imaging solutions like bioluminescence and fluorescence are useful for EV tracking studies in animal models, there is limited utility in clinical applications. Here, we present a novel EV labeling technology that enable real time, non-invasive tracking and quantitative assessment of EVs *in vitro* and *in vivo* utilizing magnetic resonance imaging (MRI). Leveraging clinically applicable magnetic agents, mesenchymal stem cells-, neural stem cells-, and amniotic fluid stem cells (AFSCs)- derived EVs were labeled directly or indirectly by labeling the secreting cell first prior to vesicle collection. The magnetic labeling did not affect the physiological characteristics of the cells, and the MR detectability of labeled-EVs were confirmed by magnetometer and *in vitro* MRI phantoms. To demonstrate the utility of MRI- assisted EV tracking, a proof of concept *in vivo* biodistribution study was conducted by injecting labeled AFSC EVs into WT and Alport mice (a model of chronic kidney disease) via different routes of administration, and tracking them via MRI at 10 min and 3 h post injection. MRI studies showed that homing of AFSC EVs to the kidney injected intra-cardiacally into Alport mice were more efficient versus the retro-orbital route, and Prussian blue staining of kidney sections confirmed the MR findings. In summary, we have developed a clinically applicable novel magnetic nanoparticle agent that can be used to label and track the biodistribution of EVs in living subjects using non- invasive, safe, and effective MRI technology that’s widely available. This technology is highly adaptable and can be deployed in both preclinical and clinical settings.

# Other: 26. Glomerular heterogeneity and modulation of miR-93-5p: the role of extracellular vesicles

**Authors:** Charmi Dedhia, Paola Aguiari, Hasmik Soloyan, Roger E De Filippo, Sargis Sedrakyan, Laura Perin

**Affiliations:** Children’s Hospital Los Angeles/University of Southern California, Los Angeles, CA, USA.

## Abstract

miRNA play important roles in the pathogenesis of renal diseases. Modulation of miRNA in podocytes and glomerular endothelial cells (GEC) has been associated with cellular damage and development of renal diseases. miR-93-5 is a potent regulator of various genes and pathways responsible of glomerular damage during pathological conditions, like VEGF, TGF and Msk2. We have evidence that miR- 93-5p is altered in the glomeruli of mice affected with X-linked Alport syndrome (AS), as well as in human AS glomeruli. In this study, we investigated the role of miR- 93-5p in glomerular cells from healthy and AS mice. We also used extracellular vesicles (EVs) derived from amniotic fluid stem cells (AFSC), which are naturally enriched with mir-93-5p to asses their therapeutic potential to rescue glomerular damage *in vitro* and *in vivo* by regulating miR-93-5p target genes. Mesangial cells, podocytes and GEC were sorted from glomeruli of male and female WT and AS mice at different stages of disease and miR-93-5p expression was evaluated by qRT-PCR. We assessed renal cortices from patients affected by AS and disease- modifying activity of human AFSC EVs by 1) applying EVs to damaged GEC and podocytes *in vitro* followed by analysis of mir-93-5p targets and 2) injecting EVs into AS mice followed by RNAseq analysis of isolated glomeruli and survival. Glomerular miR-93-5p expression differed between male and female mice and in glomerular cells throughout the progression of disease *vs*. WT. In glomerular cells from AS male mice, miR-93-5p levels were significantly lower in GEC, but not in podocytes or mesangial cells, relative to WT. Consistently, decreased miR-93-5p expression was detected in human samples from AS patients. Expression of WT1 and miR-93 in puromycin aminonucleoside damaged podocytes and expression of fibronectin and miR-93 in VEGF damaged GEC was restored to basal level in the presence of hEVs. *In vivo*, single injection of hEVs showed therapeutic effect by ameliorating the level of proteinuria and increasing life span, as shown for mouse EVs (Sedrakyan, 2017). Differential gene expression and pathway enrichment analysis showed stark differences between male and female glomeruli in WT, involving respiratory and metabolic pathways, extracellular matrix and cell adhesion molecules. In AS males, genes with functional role in lipid metabolism and angiogenic pathways were most highly regulated; AS males injected with EVs showed improved gene modulations in metabolic function and genes with functional role in the development of vasculature and angiogenesis were most highly enriched. Gender specific variation in miR-93-5p expression in glomerular cells might indicate important differences in their biology and potential response to injury during development of kidney diseases. EVs from AFSC demonstrate great potential to restore lost miR-93-5p expression in glomeruli of AS and therefore can present powerful therapeutic approach for treatment of CKD.

# Other: 27. Use of high-capacity membranes for simple, rapid exosome isolations with high yield and purity

**Authors:** Yi Zhao, Boris Levitan, Brenda Huang, Micheal Haugwitz, Andrew Farmer

**Affiliations:** Takara Bio USA, Inc, Mountain View, CA, USA.

## Abstract

**Introduction**: Despite their small size, exosomes play important roles in normal physiological processes (e.g., immune response, neuronal function, and stem cell maintenance) and diseases (e.g., cancer and liver disease). The isolation of the vesicles has historically been accomplished via ultracentrifugation. However, ultracentrifugation is time-consuming, is not scalable, requires specialized equipment, may damage vesicles during the high-speed spins, and suffers from low yield. More recently, precipitation solutions have been utilized to simplify exosome isolation protocols, but precipitation-based techniques are often inconsistent, with low yield and reduced purity. Thus, there is a significant need for a method to isolate exosomes without compromising purity or yield rapidly.

**Methods**: Here we describe the use of novel membranes conjugated to a proprietary, non- antibody based exosome-binding compound to isolate exosomes selectively. The membranes, which we have named Capturem™ membranes, have been chemically modified to have increased surface area, which allows higher binding capacity while providing highly pure and concentrated samples. Additionally, the membranes have been assembled into benchtop centrifuge-compatible spin columns, which can be used to isolate exosomes from plasma in under 30 minutes.

**Results and Conclusions**: Isolations performed with the Capturem exosome isolation spin columns produced exosomes of sizes comparable to experimental values reported in the literature; containing the key exosome protein markers CD63, CD9, and Alix; and with little or no expression of the exosome-negative markers Calnexin and Albumin. As a whole, the Capturem columns enable researchers to study exosomes to accelerate the pace of their research by obtaining high yields of noncontaminated exosomes simply and rapidly.

# Other: 28. Detection of EGFR mutations in extracellular vesicle RNA and protein corresponds to disease status in metastatic lung cancer patients

**Authors:** Emma Purcell, Sarah Owen, Emily Prantzalos, Abigail Radomski, Mina Zeinali, Nayri Carman, Nithya Ramnath, Sunitha Nagrath

**Affiliations:** ^1^Department of Chemical Engineering, University of Michigan, Ann Arbor, MI, USA.; ^2^Biointerface Institute, University of Michigan, Ann Arbor, MI, USA.; ^3^Department of Internal Medicine, University of Michigan Rogel Cancer Center, Ann Arbor, MI, USA.; ^4^Veterans Administration, Ann Arbor Healthcare System, Ann Arbor, MI, USA.

## Abstract

**Introduction**: Current cancer detection and characterization methods require a lung tissue biopsy, an invasive procedure, performed only when the patient is showing symptoms that often only arise in late-stage cancers. Extracellular vesicles offer a stable, abundant biomarker in the blood to serially profile molecular characteristics of patient tumors through a non-invasive liquid biopsy. In non-small cell lung cancer (NSCLC), identifying the presence of sensitizing and resistance epidermal growth factor receptor (EGFR) mutations informs sensitivity to tyrosine kinase inhibitors (TKIs) and dictates treatment regimes.

**Methods**: This pilot study reports the longitudinal study of EGFR mutations carried in extracellular vesicle protein (evProtein) and RNA (evRNA) to monitor metastatic non-small cell lung cancer patients. We enrolled 10 NSCLC patients: 8 with exon 19 deletion mutations and 2 with co-harbored L858R sensitizing and T790M resistance mutations. We used ultracentrifugation to isolate EVs from dilute plasma before testing the EVs for mutations based on the patient’s original tumor biopsy. In our work, we used droplet digital PCR (ddPCR) to test for mutations in the EV-RNA and western blots to test for sensitizing mutations in EV-protein.

**Results**: In our cohort of 10 NSCLC patients, mutant EV-RNA was detected in 9/10 patients. The overall detection rate for all EV-RNA mutations was 60%. Exon 19 del was detected in 7/8 patients, and in 78% of samples tested. While the detection rate of L858R remained moderate at 60%, the detection of the notoriously challenging T790M mutation was low, at 25% Sensitizing mutations were detected in 12/21 (57%) EV-Protein samples. EV-RNA and EV-Protein were then compared to clinical data and showed that increasing EV-RNA mutation burden mirrored disease progression. For 6/7 patients who were longitudinally monitored, EV-RNA mutation burden mirrored clinical trajectory. Increasing exon 19 del exoRNA burden mirrored disease progression in two patients, while decreasing burden mirrored stable disease in three patients. The three patients who saw a decrease in exon 19 del burden have remained clinically stable for an average of 192 ± 9 days after the final blood draw, while those who had progressive disease are now deceased. Of the two patients with L858R/T790M mutations, one patient’s EV-RNA burden mirrored disease trajectory, while the other did not. Conversely, EV-Protein did not appear to mirror clinical progression and was largely patient dependent.

**Conclusions**: In this novel proof of concept study, extracellular vesicles were screened for previously identified EGFR mutations carried by each patient. Changes in EV-RNA were found to correlate with disease trajectory; however, the clinical implications of EV-Protein remain unclear. This study demonstrates the potential utility of characterizing both extracellular vesicle RNA and protein cargo, allowing for multi-faceted analyses of a patient’s disease.

# Other: 29. The role of extracellular vesicles in cancer-related fatigue

**Authors:** Dilorom Sass, Wendy Fitzgerald, Leorey Saligan, Leonid Margolis

**Affiliations:** National Institute of Nursing Research, National Institute of Child Health and Human Development, USA.

## Abstract

**Purpose/Background**: Cancer Related Fatigue (CRF) is the most prevalent and distressing symptom associated with cancer and its treatments. The etiology of CRF is shown to be multifactorial, yet the exact mechanisms of CRF are poorly understood. The purpose of this study was to investigate the role of extracellular vesicles (EVs) in the inflammation mechanism of CRF. EVs are ubiquitously present in the body, exchange material between cells and can serve as biomarkers for diagnosis and treatment. No prior studies have investigated EV markers as a measure of inflammation in CRF.

**Methods**: Plasma was collected from men with non-metastatic prostate cancer receiving external beam radiation therapy (EBRT) (*n* = 40) at two time points: at the start of (T1) and 3 months after EBRT (T2). Fatigue was assessed using the Functional Assessment of Cancer Therapy - Fatigue (FACT-F). Characterization of the EVs included: size and concentrations using Nanoparticle Tracking Analysis (NTA), morphology using electron microscopy, and EV markers (CD9, CD81, TSG101) using western blot. Cytokines were measured in EV-associated and soluble (EV free) fractions using a multiplexed immunoassay system.

**Findings**: Log fold change of EV-associated analytes showed increase in Eotaxin, hsp27, IP10, MIP3α from T1 to T2 in fatigued patients compared to non-fatigued. Whereas, in soluble fraction, only survivin had a log fold increase at T2 for the fatigued cohort.

**Implications**: There is an association between EV markers and severity of fatigue in men treated with EBRT for prostate cancer. EV-associated and soluble analytes should be used for early detection of persistent CRF that can continue for months after treatment completion.

# Other: 30. Mechanistic analysis of protein transport to extracellular membrane vesicles of a hypervesiculating bacterial strain, Shewanella vesiculosa HM13

**Authors:** Tatsuo Kurihara, Kouhei Kamasaka, Chen Chen, Fumiaki Yokoyama, Tomoya Imai, Takuya Ogawa, Jun Kawamoto

**Affiliations:** ^1^Institute for Chemical Research, Kyoto University, Gokasho, Uji, Japan.; ^2^Research Institute for Sustainable Humanosphere, Kyoto University, Gokasho, Uji, Japan.

## Abstract

Shewanella vesiculosa HM13 is a hypervesiculating Gram-negative bacterium isolated from the intestine of a horse mackerel. The strain produces extracellular membrane vesicles (EMVs) that contain a single major cargo protein, P49, of unknown function. P49 is expected to be useful as a carrier to deliver foreign proteins to EMVs. To develop an EMV-based heterologous protein production system by using S. vesiculosa HM13 as the host, we analyzed the mechanism of transport of P49 to EMVs. Whole genome sequencing of S. vesiculosa HM13 revealed that the P49 gene is located downstream of a gene cluster coding for homologs of components of a type II protein secretion system (T2SS). Disruption of the genes coding for these proteins caused disappearance of P49 from EMVs. Thus, it is very likely that the T2SS-like machinery functions as a protein translocon to transport P49 to EMVs. In the vicinity of the P49 gene in the genome, we also found the genes coding for proteins that are presumably involved in the synthesis of surface glycolipids/polysaccharides. When these genes were disrupted, P49 was secreted to the extracellular space without being loaded to EMVs. The results raised the possibility that P49 is loaded onto EMVs through interaction with surface glycolipids/polysaccharides of EMVs. We further studied the transport mechanism *in vitro* by incubating purified P49 with P49-free EMVs prepared from various gene-disrupted mutants. We found that P49 was loaded onto EMVs prepared from the mutants that lack the T2SS-like machinery, whereas the loading efficiency was markedly decreased for EMVs prepared from the mutants that lack the putative surface glycolipid/polysaccharide biosynthesis genes in the P49 gene cluster. These results suggest that P49 is translocated across the outer membrane through the T2SS-like machinery and loaded onto EMVs through interaction with surface glycolipids/polysaccharides of EMVs.

# Other: 31. Mycobacterial dynamin like proteins are necessary for extracellular vesicle release in M. tuberculosis

**Authors:** Shamba Gupta, Ainhoa Palacios, Atul Khataokar, Madhuri Bhagavathula, Matthew B. Neiditch, Padmini Salgame, Rafael Prados-Rosales, G. Marcela Rodríguez

**Affiliations:** Public Health Research Institute Center and New Jersey Medical School - Rutgers, The State University of New Jersey, Newark, NJ, USA.

## Abstract

Mycobacterium tuberculosis (Mtb) secretes pathogenicity factors and immunologically active molecules via extracellular vesicles. However, little is known regarding the mechanisms and molecules involved in mycobacterial vesicle biogenesis. This study investigates molecular determinants of vesicle production in Mtb by analyzing Mtb cells under conditions of high vesicle production, such as iron restriction and cells lacking VirR. Transcriptomic analysis showed common upregulation of the iniBAC operon in association with high vesicle production in Mtb. Genetic and vesicle production analysis demonstrated that the dynamin-like proteins (DLPs) encoded by this operon, IniA and IniC, are necessary for release of extracellular vesicles by Mtb in culture and in infected macrophages. The first line antibiotic, isoniazid was found to stimulate vesicle release in a DLP-dependent manner. Extracellular vesicles purified from WT Mtb cultures or concentrated from the extracellular medium of infected macrophages stimulated release of pro- inflammatory cytokines in uninfected bone marrow-derived macrophages, whereas an iniAC mutant showed poor immunostimulatory activity. Our results provide a new understanding of the function of mycobacterial DLPs and mechanistic insights into vesicle biogenesis and enable studies that address the role of extracellular vesicles in TB pathogenesis.

# Other: 32. Intracellular delivery methods using biofunctional peptide-modified extracellular vesicles

**Authors:** Ikuhiko Nakase

**Affiliations:** Graduate School of Science, Osaka Prefecture University, Naka-ku, Sakai-shi, Japan.

## Abstract

Extracellular vesicles (exosomes, EVs) with encapsulation of biofunctional molecules (e.g., enzymes and genes) are highly expected to be next-generation therapeutic carriers because of their pharmaceutical advantages including effective usage of cell-to-cell communication routes, controlled immunogenicity, absence of cytotoxicity, brain targeting through BBB. However, methods for increasing the cellular EVs uptake efficacy must be developed to achieve effective intracellular delivery of EV contents. In this presentation, I will introduce novel techniques to enhance cellular EV uptake by modification of functional peptides on EV membranes^[1-3]^ especially using an antimicrobial protein, CAP18 derived cell- penetrating peptides^[4]^ for macropinocytosis induction and effective cellular uptake. We previously found that macropinocytosis (accompanied by actin reorganization, ruffling of the plasma membrane, and engulfment of large volumes of extracellular fluid) is important route for enhanced cellular EV uptake^[5]^. CAP18- derived sC18 peptide has high abilities for cellular uptake, and dimer-type structure of sC18 peptides, (sC18)2, shows further cell membrane penetration^[4]^. In this study, we found that (sC18)2 peptides induced macropinocytosis via glycosaminoglycan on plasma membranes of targeted cells. Furthermore, modification of the sC18 peptides or (sC18)2 peptides with stearyl-moiety on EV membranes significantly enhanced cellular EV uptake via macropinocytosis induction. In addition, ribosome-inactivating protein, saporin-artificially encapsulated EVs with modification of the (sC18)2 peptides showed glycosaminoglycan-dependent cell-killing activity. Our experimental techniques and findings are considered to contribute to the development for EV-based intracellular delivery system via macropinocytosis.

## References

[1] Nakase I, Noguchi K, Fujii I, Futaki S (2016). Vectorization of biomacromolecules into cells using extracellular vesicles with enhanced internalization induced by macropinocytosis.. Sci Rep.

[2] Nakase I, Noguchi K, Aoki A, Takatani-Nakase T, Fujii I, Futaki S (2017). Arginine-rich cell-penetrating peptide-modified extracellular vesicles for active macropinocytosis induction and efficient intracellular delivery.. Sci Rep.

[3] Nakase I, Ueno N, Katayama M (2016). Receptor clustering and activation by multivalent interaction through recognition peptides presented on exosomes.. Chem Commun (Camb).

[4] Gronewold A, Horn M, Ranđelović I (2017). Characterization of a cell-penetrating peptide with potential anticancer activity.. ChemMedChem.

[5] Nakase I, Kobayashi NB, Takatani-Nakase T, Yoshida T (2015). Active macropinocytosis induction by stimulation of epidermal growth factor receptor and oncogenic Ras expression potentiates cellular uptake efficacy of exosomes.. Sci Rep.

# Other: 33. Effects of marijuana on viral transcription in HIV-1 infected cells and resulting extracellular vesicle release

**Authors:** Catherine De Marino, Maria Cowen, Bianca Cotto, Sara Jane Ward, Ronald Tuma, Prasun Datta, Leah H. Rubin, Dianne Langford, Fatah Kashanchi

**Affiliations:** ^1^Laboratory of Molecular Virology, School of Systems Biology, George Mason University, Manassas, VA, USA.; ^2^Department of Neuroscience, Temple University, Philadelphia, PA, USA.; ^3^Department of Pharmacology, Lewis Katz School of Medicine at Temple University, Philadelphia, PA, USA.; ^4^Department of Neurology, Johns Hopkins University, Baltimore, MD, USA.

## Abstract

As of 2016, roughly 18.2 million of the approximately 36.9 million people living with HIV globally were receiving combination antiretroviral therapy (cART). Despite decades of research and development of this complex drug regimen, which is effective in the prevention of new infections, cells with an integrated HIV-1 genome have leaky transcription which can produce viral RNAs and proteins. These viral products can then be packaged into extracellular vesicles (EVs) and released from the infected cell. EVs, specifically exosomes, produced from HIV-1 infected cells contain viral mRNAs and incubation of these exosomes with cells caused a significant increase in the production of the proinflammatory cytokines, implicating EVs as a possible mechanism for the chronic inflammation observed in the CNS of people living with HIV-1 on antiretroviral therapy^[1-4]^. Previous studies have shown that marijuana use in people living with HIV is associated with a lower viral load and high CD4+ T-cell count, suggesting a potential therapeutic application. Here, we investigated the effects of cannabinoids, CBD and THC, on viral transcription in HIV-1 infected cells and resulting changes in EV release. Our data suggests CBD and THC can act as viral transcription inhibitors, potentially through two independent mechanisms. Here we show that treatment of CBD and THC on virally infected myeloids results in lowered production of intracellular and extracellular viral RNAs, such as short non-coding (TAR) and genomic (env) transcripts, as well as lowered downstream viral proteins, such as capsid protein (Pr55), cleaved capsid protein (p24) and accessory protein (Nef). Additionally, the results show a significant reduction in EVs released from infected cells. These studies are significant in that marijuana may provide a protective effect by alleviating the pathogenic effects of EVs in HIV-1 and CNS-related infections.

## References

[1] Narayanan A, Iordanskiy S, Das R (2013). Exosomes derived from HIV-1-infected cells contain trans-activation response element RNA.. J Biol Chem.

[2] Sampey GC, Saifuddin M, Schwab A (2016). Exosomes from HIV-1-infected cells stimulate production of pro-inflammatory cytokines through trans-activating response (TAR) RNA.. J Biol Chem.

[3] Barclay RA, Schwab A, DeMarino C (2017). Exosomes from uninfected cells activate transcription of latent HIV-1.. J Biol Chem.

[4] DeMarino C, Pleet ML, Cowen M (2018). Publisher correction: antiretroviral drugs alter the content of extracellular vesicles from HIV-1-infected cells.. Sci Rep.

# Other: 34. Extracellular vesicles release from infected cells prior to virion release

**Authors:** Yuriy Kim, Daniel Pinto, Gifty Mensah, Maria Cowen, James Erickson, Renaud Mahieux, Fatah Kashanchi

**Affiliations:** ^1^Laboratory of Molecular Virology, School of Systems Biology, George Mason University, Manassas, VA, USA.; ^2^CIRI International Center for Infectiology Research, Inserm U1111, CNRS UMR5308, Ecole Normale Superieure de Lyon, Université Lyon, Lyon, France.

## Abstract

Recently, it has become evident that Extracellular Vesicles (EVs) play a major role in the viral pathogenesis. Our lab has been able to elucidate the role of EVs in the pathogenesis of several different viruses including HIV-1, HTLV-1, Rift Valley Fever Virus and Ebolavirus^[1-7]^. However, timing difference between EV and virions release from infected cells has not been previously reported. We have attempted to address the spatiotemporal dynamics of EV and virions release from HIV-1 and HTLV-1 infected cells. The infected cells were synchronized in G0 quiescent stage using serum starvation. Viral latency was reversed by increasing gene expression with the addition of serum rich media and inducers (20% FBS + PMA/PHA). Supernatants and cell pellets were collected post induction at 0, 3, 6, 12, and 24 h and assayed for the presence of markers of EVs, autophagy and for the viral proteins and RNA transcripts. Results from supernatants of uninfected cells showed a peak of tetraspanin proteins (CD63, CD81, and CD9) at 6 h and a gradual decrease of all EV associated proteins by 24 h. However, the EV from HIV-1 infected cells showed all three tetraspanins present at 3 h and expression gradually increased up to 24 h. When compared to HTLV-1 infected cells, the three tetraspanin proteins peaked at 6 h and expression continued to decrease up to 24 h. HTLV-1 infected cells also showed a unique pattern of CD81 expression. Autophagy associated proteins (LC3A, LC3B and p62) from uninfected cells and HTLV-1 infected cells plateaued at 6 h, whereas in HIV-1 infected cells their expression continued to increase and peaked at 24 h. HIV-1 viral proteins (p24, gp120, Nef) expression was present at 6 h and continued to increase and peaked at 24 h. HTLV-1 proteins (p19 and gp46/61) peaked at 6 h and decreased overtime. HIV-1 and HTLV-1 gene expression was quantified, and data correlated with viral protein expression. EV release was analyzed by nanoparticle tracking analysis and significant increase of EV concentration overtime in both uninfected and infected samples was observed. Finally, virus rescue assay with the use of naïve cells was performed on 6- and 24-h supernatants. HIV-1 supernatant from 6-h sample was found not to be infectious, however the virus from 24-h sample was rescued. Our data indicates that EV release may occur prior to virion release from infected cells. The released EVs can be implicated in the facilitation of virus spread and deleterious effect on the naive recipient cells.

## References

[1] Narayanan A, Iordanskiy S, Das R (2013). Exosomes derived from HIV-1-infected cells contain trans-activation response element RNA.. J Biol Chem.

[2] Sampey GC, Saifuddin M, Schwab A (2016). Exosomes from HIV-1-infected cells stimulate production of pro-inflammatory cytokines through trans-activating response (TAR) RNA.. J Biol Chem.

[3] Ahsan NA, Sampey GC, Lepene B (2016). Presence of viral RNA and proteins in exosomes from cellular clones resistant to rift valley fever virus infection.. Front Microbiol.

[4] Barclay RA, Schwab A, DeMarino C (2017). Exosomes from uninfected cells activate transcription of latent HIV-1.. J Biol Chem.

[5] DeMarino C, Pleet ML, Cowen M (2018). Antiretroviral drugs alter the content of extracellular vesicles from HIV-1-infected cells.. Sci Rep.

[6] Pleet ML, Erickson J, DeMarino C (2018). Ebola virus VP40 modulates cell cycle and biogenesis of extracellular vesicles.. J Infect Dis.

[7] Pinto DO, DeMarino C, Pleet ML (2019). HTLV-1 extracellular vesicles promote cell-to-cell contact.. Front Microbiol.

# Other: 35. Separation of EVs from virions in coronavirus infections

**Authors:** James Erickson, Maria Cowen, Yuriy Kim, Anoop K. Pal, Heather Branscome, Archana Gupta, Fatah Kashanchi

**Affiliations:** ^1^Laboratory of Molecular Virology, School of Systems Biology, George Mason University, Manassas, VA, USA.; ^2^Izon Science (IZON), Medford, MA, USA.; ^3^American Type Culture Collection (ATCC), Manassas, VA, USA.; ^4^Systems Biosciences (SBI), Palo Alto, CA, USA.

## Abstract

Since the severe acute respiratory syndrome corona virus 2 (SARS-CoV-2) was declared a pandemic in mid-March of 2020 by the World Health Origination (WHO), laboratories around the world started research into diagnostics, therapeutics, and treatments^[1]^. In recent years, the importance of extracellular vesicles (EVs) in the pathogenesis of viral infections have been found in the cases of many viral pathogens including few DNA and RNA viruses including human T-cell leukemia virus-1 (HTLV-1) and human immunodeficiency virus-1 (HIV-1)^[2,3]^. EVs from HIV-1 infected cells on uninfected macrophages induces an increase in the proinflammatory cytokines^[3]^. While EVs from HTLV-1 infected cells on uninfected recipient cells promoted the localization and cellular contact by cells, this directly influences the pathogenesis of HTLV-1 as the virus mainly infects other cells by cell to cell contact^[2]^. Similar to retroviruses, coronaviruses are also positive strand RNA viruses, except they replicate in the cytoplasm and may regulate chromosomal DNA depending on the strain of virus. We have recently began working on beta- coronaviruses, including OC43 (BSL2 strain) and SARS-CoV-2 (BSL3 strain). Our initial experiments focus on isolation of EVs away from virions using either an iodixanol gradients or Izon sizing columns. We have successfully separated the two from one another mainly due to their density and potentially size differences. We found that EVs from multiple coronaviruses are not infectious and viral particles treated with UV irradiation are also not infectious. We also have found that coronavirus EVs caused T-cell death, which may corelate with lymphopenia observed in COVID patients. Along these lines coronavirus EVs can activate other viral genes (i.e., HIV-1 or HTLV-1) when these genes are integrated into the genome, further implying that these EVS regulate chromosomal gene expression. Finally, the mechanism(s) of how these EVs may cause such diverse effects on T-cells and other viral gene expression will be discussed.

## References

[1] Cucinotta D, Vanelli M (2020). WHO Declares COVID-19 a Pandemic.. Acta Biomed.

[2] Pinto DO, DeMarino C, Pleet ML (2019). HTLV-1 extracellular vesicles promote cell-to-cell contact.. Front Microbiol.

[3] Sampey GC, Saifuddin M, Schwab A (2016). Exosomes from HIV-1-infected cells stimulate production of pro-inflammatory cytokines through trans-activating response (TAR) RNA.. J Biol Chem.

# Other: 36. Reduction of the therapeutic dose of silencing RNA by packaging it in extracellular vesicles via a pre-microRNA backbone

**Authors:** Ryan Reshke, James A. Taylor, Alexandre Savard, Huishan Guo, Luke H. Rhym, Piotr S. Kowalski, My Tran Trung, Charles Campbell, Daniel G. Anderson, Derrick Gibbings

**Affiliations:** Department of Cellular and Molecular Medicine, University of Ottawa, Ottawa, Canada.; The Koch Institute for Integrative Cancer Research, Massachusetts Institute of Technology, Cambridge, MA, USA.

## Abstract

Current delivery vehicles enable less than 1% of silencing RNA (siRNA) cargoes to escape into the cytoplasm. This necessitates high doses in patients that have demonstrated toxicity and constrained use of siRNA therapeutics to targets expressed in liver. Small extracellular vesicles (sEVs) are naturally released from virtually all cell types and can traffic RNA between cells, and are touted as a bio- inspired delivery vehicle. However, doses of siRNA used to suppress targets in many published studies using sEVs far exceed those of other delivery vehicles. This suggests that sEVs are quantitatively poor at delivering cargoes into target cells and questions the model in which the principal role of sEVs is intercellular delivery of cargoes. We demonstrate that sEVs naturally contain thirty copies or less of specific miRNA per sEV. Nonetheless, pre-miR-451 derivatives are enriched by 1,000-fold in sEVs produced by many cell types. Reprogramming the unique Dicer- independent pre-miR-451 secondary structure with new siRNA sequences enables robust siRNA enrichment in sEVs and these sEVs reduce siRNA target expression in mouse liver, intestine and kidney podocytes with doses of siRNA that are at least 10-fold lower than lipid nanoparticles. The capacity of sEVs to deliver siRNA is abrogated when their membranes are disrupted by electroporation. This demonstrates that intact sEVs can be highly efficient at RNA delivery and provides an RNA stem-loop to harness this by enabling robust, scalable packaging of siRNA into sEVs.

# Other: 37. Cell type-specific and disease-associated protein networks in extracellular vesicles isolated from human iPSC-derived neural cells and Alzheimer’s disease brain tissues

**Authors:** Yang You, Satoshi Muraoka, Mark P. Jedrychowski, Jianqiao Hu, Amanda K. McQuade, Tracy Young-Pearse, Roshanak Aslebagh, Scott A. Shaffer, Mathew Blurton-Jones, Wayne W. Poon, Steven P. Gygi, Tsuneya Ikezu

**Affiliations:** ^1^Department of Pharmacology & Experimental Therapeutics, Boston University School of Medicine, Boston, MA, USA.; ^2^Department of Cell Biology, Harvard Medical School, Boston, MA, USA.; ^3^Department of Neurobiology and Behavior, Institute for Memory Impairments and Neurological Disorders, University of California, Irvine, CA, USA.; ^4^Department of Neurology, Brigham and Women’s Hospital, Harvard Medical School, Boston, MA, USA.; ^5^Department of Biochemistry and Molecular Pharmacology, University of Massachusetts Medical School, Worcester, MA, USA.; ^6^Mass Spectrometry Facility, University of Massachusetts Medical School, Shrewsbury, MA USA.; ^7^Department of Neurology, Alzheimer’s Disease Center, Boston University School of Medicine, Boston, MA, USA.; ^8^Center for Systems Neuroscience, Boston University, Boston, MA, USA.

## Abstract

Extracellular vesicles (EVs) have gathered great interest in studying neurodegenerative diseases with the capability of transferring pathogenic molecules and the source of liquid biopsies. Here we performed combined label-free and tandem mass tag-labeling based quantitative mass-spectrometry of EVs isolated from human induced pluripotent stem cells (hiPSCs) and Alzheimer’s disease (AD) brain tissues to conduct a comprehensive EV proteomics study on AD. Cell type-specific EV protein signatures were identified from hiPSC-derived excitatory neurons, astrocytes, microglia-like cells and oligodendrocytes. Furthermore, a whole protein co-expression network analysis identified a module most significantly associated with AD pathology and cognitive function and enriched in astrocytic markers, particularly reactive astrocytes. Proteins within this module were regulated by pro-inflammatory molecules for the inflammatory processes. Our study presents unique human neural cell type-specific EV markers and their application for liquid biopsy-based EV AD biomarkers and disease monitoring.

# Other: 38. Exosomes mediate Zika virus transmission through SMPD3 neutral Sphingomyelinase in cortical neurons

**Authors:** Hameeda Sultana

**Affiliations:** Old Dominion University, Norfolk, VA, USA.

## Abstract

The transmission dynamics of ZIKA virus (ZIKV) in or between neurons, or within the developing brains of the fetuses are not fully understood. Using primary cultures of murine cortical neurons, we show that ZIKV uses exosomes as mediators of viral transmission between neurons. Increased production of exosomes from neuronal cells was noted upon ZIKV infection. Neuronal exosomes contained both ZIKV viral RNA and protein(s) that were highly infectious to naïve cells. RNaseA and neutralizing antibodies treatment studies suggested presence of viral RNA/proteins inside exosomes. Exosomes derived from time- and dose-dependent incubations showed increasing viral loads suggesting higher packaging and delivery of ZIKV RNA and proteins. Furthermore, we noted that ZIKV induced both activity and gene expression levels of neutral Sphingomyelinase (nSMase)-2/SMPD3, an important molecule that regulates production and release of exosomes. Silencing of SMPD3 in neurons resulted in reduced viral burden and transmission through exosomes. Treatment with SMPD3 specific inhibitor GW4869, significantly reduced ZIKV loads in both cortical neurons and in exosomes derived form these neuronal cells. Taken together, our results suggests that ZIKV modulates SMPD3 activity in cortical neurons for its infection and transmission through exosomes perhaps leading to severe neuronal death that may result in neurological manifestations such as microcephaly in the neonatal developing embryonic brains and other complications associated with Guillain-Barré syndrome in adults.

# Other: 39. Understanding Intracellular Fate of EV-delivered Content

**Authors:** Killian P. O’Brien, Stefano Ughetto, Xandra O. Breakefield

**Affiliations:** Department of Neurology, Massachusetts General Hospital, Boston, MA, USA.

## Abstract

Despite much work performed on evaluating the potential effects of extracellular vesicles (EVs), the functional uptake of their cargo is still controversial. This project aimed to demonstrate that EV content (protein and mRNA) is protected and can be subsequently transferred with functional activity into recipient cells, while also developing a tool to assess and quantify functional EV uptake.

**Methods:** Fusion proteins used were mitochondrial localized coxVIII-CFP-nanoluc(Cox) and nuclear localized H2B-RFP-nanoluc(H2B).

**Results:** HEK293T cell-derived EVs protected Cox proteins from proteinase K digestion while demonstrating significantly improved efficiency of uptake when compared to free protein, as measured by bioluminescence that was still detectable in recipient cells 96 hrs post EV-exposure. To confirm functional uptake, recipient cells exposed to EVs containing H2B for 72 hrs were imaged and some recipient cells manifested fluorescent red nuclei. To demonstrate the presence of functional mRNA within EVs, producer cells were transfected for such a duration as not to have detectable levels of protein in the EVs while still containing detectable levels of mRNA (qPCR) even after RNaseA treatment. Transfer of these EVs to HeLa cells showed an increase in expression of H2B which was blocked by cyclohexamide, confirming translation of the mRNA (2.2 kb). To determine if recycling of EV delivered proteins occurs, recipient HeLa cells were exposed to EVs containing Cox for 72 hrs. All extracellular EVs were removed and cells were trypsinized (0.25% for 30 min) to remove any non-internalized Cox protein. 48 hrs later, EVs (CD63+ and CD9+) released from cells contained Cox suggesting recycling of protein or possibly recycling of entire EVs. Lastly, an assay was developed to measure functional EV uptake. Nanoluc protein was split in two and fused to mTurquoise2(N65) or mScarlet-I(66C). Expression of each fragment alone exhibited non-detectable levels of luminescence while expressing both together had a significantly increased signal. Delivery of either fragment within an EV to a cell expressing the corresponding fragment worked as confirmation and quantification of EV uptake (HEK293, U87, HeLa cells).

**Summary/Discussion:** This study robustly demonstrates EV delivery of functional mRNA and protein to cells, while also establishing a simple assay to quantify and validate functional EV uptake.

# Other: 40. Smart Microprobes Imbued with Recognition Element as a Sensitive Bioanalysis Platform for Exosomes

**Authors:** Chukwumaobim D. Nwokwu, Saif Mohammad Ishraq Bari, Gergana G. Nestorova

**Affiliations:** ^1^Molecular Science and Nanotechnology, Louisiana Tech University, Ruston, LA, USA.; ^2^Micro and Nanoscale Systems Engineering, Louisiana Tech University, Ruston, LA, USA.; ^3^School of Biological Sciences, Louisiana Tech University, Ruston, LA, USA.

## Abstract

Circulating exosomes have become useful biomarkers for precise and noninvasive diagnosis and disease monitoring. However, sample purity is a drawback for current liquid-phase methods for exosome isolation. We report a selective solid-phase technology for isolation of pure exosome populations. Microneedles (300 μm × 30 mm) were functionalized with exosome-specific anti-CD63 antibodies and their capture efficiency assessed via Fluorocet assay post-incubation in astrocyte-derived exosome suspension (EXO) enriched by a standard kit, as well as direct incubation in conditioned astrocyte medium (CAM), while blocking non-specific binding with 0.1% BSA in PBS. Our results indicated a 6-fold increase in exosomes captured by microprobes incubated overnight on ice in EXO (23 × 106 exosomes/probe) vis-à-vis 2 h incubation (3.0 × 106 exosomes/probe), and 2 folds more than the overnight probes at room temperature (12.9 × 106 exosomes/probe). The microprobe’s exosome loading capacity decreased when incubated in conditioned astrocyte medium, indicating that longer incubation at lower temperatures in enriched exosome suspension favors more efficient exosome capture. Our designed probe was also amenable to exosomal protein and miRNA extraction, in amounts sufficient for downstream analyses. Future works will focus on its integration into a lab-on-a-chip platform.

# Other: 41. Extracellular vesicles for drug delivery to the brain

**Authors:** Bhagyashree S. Joshi, Marit A. De Beer, Ben N.G. Giepmans, Sameh A. Youssef, Alain de Bruin, Harm H. Kampinga, Inge S. Zuhorn

**Affiliations:** ^1^University of Groningen, University Medical Center Groningen, Department of Biomedical Engineering, Groningen, The Nederlands.; ^2^University of Groningen, University Medical Center Groningen, Department of Biomedical Sciences of Cells and Systems, Groningen, The Nederlands.; ^3^University of Utrecht, Department of Biomolecular Health Sciences, Utrecht, The Nederlands.

## Abstract

Extracellular vesicles have advantageous properties for drug delivery applications, including non-immunogenicity and homing capacity. Important parameters for efficient drug delivery to the brain using (natural) nanocarriers are efficient crossing of the blood-brain barrier (BBB) and spatiotemporal control of cargo release.

Here we show that EVs derived from neural stem cells (NSCs) are taken up by brain microvascular endothelial cells following binding to heparan sulfate proteoglycans, and efficiently cross the BBB^[1]^.

In addition, we developed an analytical methodology, combining state-of-the-art molecular tools and correlative light and electron microscopy to demonstrate that EV cargo release occurs from endosomes/lysosomes^[2]^.

Finally, we have explored the potential of neural stem cell derived EVs enriched with DNAJB6 as a therapeutic intervention for Huntington’s disease (HD). HD is a neurodegenerative disorder characterized by aggregation of the huntingtin (HTT) protein containing expanded polyglutamine (polyQ) tracts. DNAJB6, a DNAJ chaperone, has been reported to efficiently inhibit polyQ aggregation *in vitro* in cell models, and *in vivo* in HD animal models^[3]^. Administration of DNAJB6-containing EVs to cells expressing expanded polyQ tracts suppressed HTT aggregation. Furthermore, intrathecal injection of DNAJB6-enriched EVs into R6/2 transgenic HD mice significantly reduced mutant HTT aggregation in the brain^[4]^. Taken together, our data suggest that EV-mediated molecular chaperone delivery may be an effective way to reduce polyQ aggregation and potentially treat polyQ diseases, including HD.

## References

[1] Joshi BS, Zuhorn IS (2020). Heparan sulfate proteoglycan-mediated dynamin-dependent transport of neural stem cell exosomes in an in vitro blood-brain barrier model.. Eur J Neurosci..

[2] Joshi BS, de Beer MA, Giepmans BNG (2020). Endocytosis of Extracellular Vesicles and Release of Their Cargo from Endosomes.. ACS Nano..

[3] Kakkar V, Månsson C, de Mattos EP (2016). The S/T-Rich Motif in the DNAJB6 Chaperone Delays Polyglutamine Aggregation and the Onset of Disease in a Mouse Model.. Mol Cell..

[4] Joshi BS, Youssef SA, Bron R DNAJB6b-enriched exosomes decrease polyglutamine aggregation in in vitro and in vivo models of Huntington’s disease..

# Other: 42. The uptake, trafficking, and biodistribution of Bacteroides thetaiotaomicron (Bt) generated outer membrane vesicles

**Authors:** Simon Carding

**Affiliations:** Gut Microbes and Health, Quadram Institute Bioscience, Norwich Research Park, Norwich United Kingdom.; Norwich Medical School, University East Anglia, Norwich, United Kingdom.

## Abstract

The human gastrointestinal tract is home to hundreds of trillions of microorganisms (the microbiome) that perform a vital role in food digestion and providing essential nutrients and vitamins. They also play an important role in metabolizing medicines and drugs, and in resisting infection by pathogens. Gut microbes are however susceptible to change with alterations in their makeup and activity occurring as a result of exposure to various environmental factors such as diet, drugs, pathogens and behaviour. Such changes have been associated with more than 90% of human diseases affecting the gut, liver, joints, heart and brain. A central question to discriminating between association and causality is, how do gut microbes communicate with their host to affect physiological changes at the cellular and organ level within the gut and beyond? We have uncovered roles for microbiota-derived metabolites and highly stable, nanosized microvesicles (bacterial extracellular vesicles; BEVs) naturally produced in the gut by prominent members of the intestinal microbiota in cross-kingdom communication. We have shown that specific bacteria and their metabolites can affect various sensory cells of the immune, endocrine and nervous systems within the intestinal mucosa and that BEVs can bring about changes in host cell physiology by delivering various cargo including metabolic enzymes and mediators of intracellular signaling. Via their ability to cross the intestinal and respiratory epithelium and access the lymphatic and vascular system they can activate innate and adaptive immune cells locally and throughout the body to promote local and systemic regulatory immune responses. Furthermore, we have exploited this BEV-mediated cross-kingdom communication pathway to develop a biologics delivery technology platform using BEVs to deliver therapeutic proteins and vaccine antigens directly to mucosal tissues. Pre-clinical studies highlight the utility of this technology in both boosting natural immunity and in preventing and treating infection and autoimmune mediated pathologies that affect the gut and lungs, and other tissues.

# Other: 43. Infectious Exosomes/Microvesicles in Degenerative and Neoplastic Stem Cell Pathologies

**Authors:** Dennis A. Steindler

**Affiliations:** The UNC Eshelman School of Pharmacy and Institute for Innovation, the University of North Carolina Chapel Hill, Chapel Hill, NC, USA.; Steindler Consulting, Boston, FA, USA.

## Abstract

The role of exosomes/microvesicles (“EMVs”) in cell-cell communication is important in understanding infectious-like transmission of disease in everything from COVID-19 to Parkinson’s and cancer. Our previous studies have linked infectious disease to neurodegenerative diseases and cancer^[1]^, where stem cells have been shown to have a vulnerability to EMV conveyance of disease-associated nucleic acids and proteins^[2,3]^. We also showed^[4]^ that lectins, toxins and viruses have the ability to bind to particular glycoconjugates on the surfaces of stem and differentiated neural cells, and transcellularly transport to distant central nervous system (“CNS”) sites. This hijacking of the neural connectome leads to stem cell pathologies where not enough differentiated progeny are generated and potentially contribute to neurodegenerative disease, or, on the contrary, lead to too many progeny being generated as in the case of glioblastoma^[5,6]^, where the transcellular transport of such potentially infectious EMV molecular cargoes may underlie gliomagenesis and spread^[7]^. We have been studying such an infectious nature of Parkinson’s disease by way of characterizing stem cell EMVs from normal, idiopathic, gene-identified (e.g., LRRK2 G2019S mutant) and gene-corrected Parkinson’s Disease patients using Nanosight technology, immunocytochemistry and gene expression profiling to identify at-risk networks involved in the initiation and propagation of disease. We have identified EMV-associated genes including SOD1, SOD2, HIF1a, APP, JAK2 and GSK3B involved in both stem cell behavior and neurodegeneration, and furthermore showed that LRRK2 gene correction altered the molecular profile of EMVs to a near normal expression pattern as seen in control iPSC-derived dopamine neuron EMVs. Knowing the cell and molecular bases for route of entry and system to system transmission of pathogenic elements will help us to better design precision therapies that can target particular virus-cell, cell-cell and multisystem interactions underlying standard brain versus disease-associated neural functions. For example, the highly pathogenic H5N1 virus has been shown to enter the CNS^[8]^, potentially from peripheral, e.g., vagus nerve innervation of primary tissue infection sites including the lungs and GI system, and can lead to a CNS Parkinsonian phenotype. With evidence growing for neurotropism of the SARS-CoV-2 coronavirus and potentially for many other emerging viruses, it is important to determine the precise mechanisms by which infectious agents and EMVs may contribute to latent disease, including neurodegenerative diseases and cancers that may arise later in life^[9]^.

## References

[1] Ngô HM, Zhou Y, Lorenzi H (2017). Toxoplasma Modulates Signature Pathways of Human Epilepsy, Neurodegeneration & Cancer.. Sci Rep.

[2] Candelario KM, Steindler DA (2014). The role of extracellular vesicles in the progression of neurodegenerative disease and cancer.. Trends Mol Med.

[3] Candelario KM, Balaj L, Zheng T (2020). Exosome/microvesicle content is altered in leucine-rich repeat kinase 2 mutant induced pluripotent stem cell-derived neural cells.. J Comp Neurol.

[4] Steindler DA, Cooper NG (1986). Wheat germ agglutinin binding sites in the adult mouse cerebellum: light and electron microscopic studies.. J Comp Neurol.

[5] Ignatova TN, Kukekov VG, Laywell ED (2002). Human cortical glial tumors contain neural stem-like cells expressing astroglial and neuronal markers in vitro.. Glia.

[6] Sood D, Tang-Schomer M, Pouli D (2019). 3D extracellular matrix microenvironment in bioengineered tissue models of primary pediatric and adult brain tumors.. Nat Commun.

[7] Skog J, Würdinger T, van Rijn S (2008). Glioblastoma microvesicles transport RNA and proteins that promote tumour growth and provide diagnostic biomarkers.. Nat Cell Biol.

[8] Jang H, Boltz D, Sturm-Ramirez K (2009). Highly pathogenic H5N1 influenza virus can enter the central nervous system and induce neuroinflammation and neurodegeneration.. Proc Natl Acad Sci USA.

[9] Ngô HM, Zhou Y, Lorenzi H (2017). Toxoplasma Modulates Signature Pathways of Human Epilepsy, Neurodegeneration & Cancer.. Sci Rep.

# Other: 44. Tracking exosomes *in vitro* and *in vivo*

**Authors:** Frederik J. Verweij, Maarten Bebelman, Graça Raposo, D. Michiel Pegtel, Guillaume van Niel

**Affiliations:** ^1^Institute for Psychiatry and Neurosciences of Paris, Hopital Saint-Anne, Université de Paris, INSERM U1266, Paris, France.; ^2^Institut Curie, PSL Research University, CNRS, UMR144, Paris, France.; ^3^Department of Pathology, Cancer Center Amsterdam, Amsterdam University Medical Center, Amsterdam, The Netherlands.; ^4^Division of Medicinal Chemistry, Amsterdam Institute for Molecules Medicines and Systems, VU University, Amsterdam, The Netherlands.

## Abstract

Exosomes are a subclass of Extracellular Vesicles able to elicit a phenotypic response in target cells, and correspond to the intraluminal vesicles (ILVs) of multivesicular endosomes (MVEs) formed during endosome maturation. Before these MVEs fuse with the plasma membrane - the final step of exosome biogenesis - they undergo dynamic interactions with other endosome-subtypes and organelles that will impact the dynamics of exosome secretion. After secretion, they face the complex 3D environment of the body, before being taken up by target cells. Due to a lack of appropriate model systems, it remains challenging to understand how factors impact their physiology *in vivo*. We developed *in vitro* and *in vivo* model systems to study exosome dynamics and function by expressing CD63-pHluorin in cells and zebrafish embryos^[1-3]^. This work represents a comprehensive and complimentary approach to study endogenous EV regulation and function *in vitro* and *in vivo* at high spatiotemporal accuracy.

## References

[1] Verweij FJ, Bebelman MP, Jimenez CR (2018). Quantifying exosome secretion from single cells reveals a modulatory role for GPCR signaling.. J Cell Biol.

[2] Verweij FJ, Revenu C, Arras G (2019). Live Tracking of Inter-organ Communication by Endogenous Exosomes In Vivo.. Dev Cell.

[3] Bebelman M.P., Bun P., Huveneers S. (2020). Real-time imaging of multivesicular body–plasma membrane fusion to quantify exosome release from single cells.. Nat Protoc.

# Other: 45. A CRISPR-Cas9-based reporter system for single-cell detection of extracellular vesicle-mediated functional transfer of RNA

**Authors:** Olivier G. de Jong, Daniel E. Murphy, Imre Mäger, Eduard Willms, Antonio Garcia-Guerra, Jerney J. Gitz-Francois, Juliet Lefferts, Dhanu Gupta, Sander C. Steenbeek, Jacco van Rheenen, Samir El Andaloussi, Raymond M. Schiffelers, Matthew J. A. Wood, Pieter Vader

**Affiliations:** ^1^Laboratory of Clinical Chemistry and Hematology, University Medical Center Utrecht, Utrecht, The Netherlands.; ^2^Department of Paediatrics, University of Oxford, Oxford, United Kingdom.; ^3^Department of Physiology, Anatomy and Genetics, University of Oxford, Oxford, United Kingdom.; ^4^Department of Physics, University of Oxford, Oxford, United Kingdom.; ^5^Pediatric Pulmonology and Regenerative Medicine Center, University Medical Center Utrecht, Utrecht, The Netherlands.; ^6^Department of Laboratory Medicine, Clinical Research Center, Karolinska Institutet, Huddinge, Sweden.; ^7^Department of Molecular Pathology, Oncode Institute, Netherlands Cancer Institute, Amsterdam, The Netherlands.; ^8^Department of Experimental Cardiology, University Medical Center Utrecht, Utrecht, The Netherlands.

## Abstract

Extracellular vesicles (EVs) play an important role in communication between cells through functional transfer of bioactive cargo, including RNA. Despite increasing interest in EV-mediated RNA transfer, in-depth knowledge on mechanisms underlying EV-mediated RNA delivery and processing is limited due to a lack of suitable readout systems. Here, we report a novel CRISPR/Cas9-based reporter system that allows for studying EV-mediated RNA transfer at a single-cell level. After validation of this system by studying the role of known targets involved in EV uptake and intracellular membrane trafficking, we have employed this system to uncover various novel genes that play a regulatory role in functional RNA transfer. Thus, this novel system may be used for the study of specific genetic targets and pathways underlying EV-mediated functional RNA delivery.

# Other: 46. Exploitation of the Leishmania Exosomal Pathway by Leishmania RNA virus 1

**Authors:** Martin Olivier

**Affiliations:** The Research Institute of the McGill University Health Centre, Montréal, Canada.

## Abstract

Leishmaniasis, a complex pattern of diseases caused by sand fly-transmitted Leishmania sp. causes over 2 million new infections and 30,000 deaths each year. In mammals, Leishmania parasites establish a persistent infection by inducing MØ dysfunction through direct manipulation of MØ signaling. We have deciphered the mechanisms whereby Leishmania exploits MØ signaling pathways to block microbicidal functions and innate inflammatory responses during infection. Work from my lab discovered that Leishmania major GP63 was enriched in Leishmania exosomes and to play a pivotal role in those deactivation process of MØ responses.

We reported that Leishmania exosomes are released in the gut of its sand fly vector and co-inoculated with Leishmania promastigotes during blood meals. Co-egested Leishmania exosomes were found to exacerbate cutaneous leishmaniasis skin lesions by overproducing inflammatory cytokines fueling Th17 immune response.

Recently, Leishmania RNA virus 1 (LRV1) infecting certain Leishmania species was found to be associated with aggressive mucocutaneous disease triggered in response to this dsRNA virus. However, it was unclear how LRV1 is exposed to the mammalian host cells. In higher eukaryotes, some viruses are known to utilize the host exosome pathway for their formation and cell-to-cell spread. As a result, exosomes derived from infected cells contain viral material or particles. Recently, we found that LRV1 exploits the Leishmania exosome pathway to reach the extracellular environment. Biochemical and electron microscopy analyses of exosomes derived from LRV1-infected Leishmania revealed that most dsRNA LRV1 co-fractionated with exosomes, and that a portion of viral particles was surrounded by these vesicles. Transfer assays of LRV1-containing exosome preparations showed that a significant number of parasites were rapidly and transiently infected by LRV1. Remarkably, these freshly infected parasites generated more severe lesions in mice than non-infected ones. Moreover, mice co-infected with parasites and LRV1-containing exosomes also developed a more severe disease. Overall, this work provided evidence that Leishmania exosomes function as viral envelopes, thereby facilitating LRV1 transmission and increasing infectivity in the mammalian host.

# Other: 47. Delivery of pre-miRNA Cargo to Tumor Microvesicles

**Authors:** Crislyn D’Souza-Schorey, James Clancy, Ye Zhang

**Affiliations:** Department of Biological Sciences, University of Notre Dame, Notre Dame, IN, USA.

## Abstract

The regulated shedding of extracellular vesicles (EVs) is now understood to serve as an important means of intercellular communication. Tumor-derived microvesicles (TMVs) comprise a class of extracellular vesicles released from tumor cells that are now understood to facilitate communication between the tumor and the surrounding microenvironment. Despite their significance, the regulation and mechanisms governing the trafficking of bioactive cargos, including microRNAs (miRNAs), to TMVs at the cell surface remain poorly defined. While miRNA recruitment ito exosomes has been reported, including the sequence specific mechanisms for targeting miRNA, current understanding of miRNA loading into TMVs has only recently begun to be elucidated. We have described a molecular pathway for the delivery of miRNA cargo to nascent TMVs involving the dissociation of a pre-miRNA/Exportin-5 complex from Ran-GTP following nuclear export and its subsequent transfer to a cytoplasmic shuttle comprised of ARF6-GTP and GRP1. As such, ARF6 activation increases the pre-miRNA cargo contained within TMVs via a process that requires casein kinase 2-mediated phosphorylation of Ran-GAP1. Further, TMVs were found to contain pre-miRNA processing machinery including Dicer and Argonaute 2, which allow for cell-free pre-miRNA processing within shed vesicles. These findings offer cellular targets to block the loading and processing of pre-miRNAs within TMVs. As with the exosomes, it is possible that as we continue to clarify the heterogeneity of microvesicle populations we will similarly decode additional mechanisms of miRNA sorting to microvesicles.

## References

[1] Clancy J.W., Zhang Y., Sheehan C. (2019). An ARF6 - Exportin-5 axis delivers pre-miRNA cargo to tumour microvesicles.. Nat Cell Biol.

# Other: 48. Head and Neck Cancer Exosomes Drive MicroRNA-mediated Reprogramming of Local Neurons

**Authors:** Patrick J. Hunt, Moran Amit

**Affiliations:** ^1^Medical Scientist Training Program, Baylor College of Medicine, Houston, TX, USA.; ^2^Department of Neurosurgery, Division of Surgery, The University of Texas MD Anderson Cancer Center, Houston, TX, USA.; ^3^Department of Head and Neck Surgery, Division of Surgery, The University of Texas MD Anderson Cancer Center, Houston, TX, USA.

## Abstract

Solid tumors are complex collections of cells surrounded by benign tissues that both influence and are influenced by the tumor. These surrounding cells include vasculature, immune cells, neurons, and other cell types that are collectively known as the tumor microenvironment. Tumors manipulate their microenvironment for the benefit of the tumor. Autonomic neurons innervate and drive malignant growth in a variety of solid tumors. However, the mechanisms by which these neuron-tumor relationships are formed have not been well understood. Recently, Amit et al. described that trophic relationships between oral cavity squamous cell carcinomas (OCSCCs) and nearby autonomic neurons arise through direct signaling between tumors and local neurons. An inducible tumor model in which 4NQO was introduced into the drinking water of *Trp53* knockout mice was used to model OCSCC-microenvironment interactions. Using this model, this group discovered that loss of p53 expression in OCSCC tumors resulted in increased nerve density within these tumors. This neuritogenesis was controlled by tumor-derived microRNA-laden extracellular vesicles (EVs). Specifically, EV-delivered miR-34a inhibited neuritogenesis, whereas miR-21 and miR-324 increased neuritogenesis. The neurons innervating p53-deficient OCSCC tumors were predominantly adrenergic and arose through the transdifferentiation of trigeminal sensory nerve fibers to adrenergic nerve fibers. This transdifferentiation corresponded with increased expression of neuron-reprogramming transcription factors, including POU5F1, KLF4, and ASCL1, which were overexpressed in the p53-deficient samples, and are proposed targets of miR-34a-mediated regulation. Human OCSCC samples enriched in adrenergic neuron markers are associated strongly with poor outcomes, thus demonstrating the relevance of these findings in cancer patients.
